# Less Cytotoxic Protoflavones as Antiviral Agents: Protoapigenone 1′-*O*-isopropyl ether Shows Improved Selectivity Against the Epstein–Barr Virus Lytic Cycle

**DOI:** 10.3390/ijms20246269

**Published:** 2019-12-12

**Authors:** Máté Vágvölgyi, Gábor Girst, Norbert Kúsz, Sándor B. Ötvös, Ferenc Fülöp, Judit Hohmann, Jean-Yves Servais, Carole Seguin-Devaux, Fang-Rong Chang, Michael S. Chen, Li-Kwan Chang, Attila Hunyadi

**Affiliations:** 1 Institute of Pharmacognosy, Interdisciplinary Excellence Centre, University of Szeged, 6720 Szeged, Hungary; vagvolgyi.mate@pharm.u-szeged.hu (M.V.); girst.gabor@pharmacognosy.hu (G.G.); hohmann@pharm.u-szeged.hu (J.H.); 2 Institute of Pharmaceutical Chemistry, University of Szeged, 6720 Szeged, Hungary; otvossandor@pharm.u-szeged.hu (S.B.Ö.); fulop@pharm.u-szeged.hu (F.F.); 3 MTA-SZTE Stereochemistry Research Group, Hungarian Academy of Sciences, 6720 Szeged, Hungary; 4 Interdisciplinary Centre for Natural Products, University of Szeged, 6720 Szeged, Hungary; 5 Department of Infection and Immunity, Luxembourg Institute of Health, L-4354 Esch-sur-Alzette, Luxemburg; Jean-Yves.Servais@lih.lu (J.-Y.S.); Carole.Devaux@lih.lu (C.S.-D.); 6 Graduate Institute of Natural Products, Kaohsiung Medical University, Kaohsiung 807, Taiwan; aaronfrc@kmu.edu.tw; 7 Department of Biochemical Science and Technology, College of Life Science, National Taiwan University, Taipei City 10617, Taiwan; b05b02041@ntu.edu.tw (M.S.C.); changlk@ntu.edu.tw (L.-K.C.)

**Keywords:** natural product, drug discovery, protoflavonoid, continuous-flow chemistry, oxime, antitumor, antiviral, Epstein–Barr virus, lytic cycle

## Abstract

Protoflavones, a rare group of natural flavonoids with a non-aromatic B-ring, are best known for their antitumor properties. The protoflavone B-ring is a versatile moiety that might be explored for various pharmacological purposes, but the common cytotoxicity of these compounds is a limitation to such efforts. Protoapigenone was previously found to be active against the lytic cycle of Epstein–Barr virus (EBV). Further, the 5-hydroxyflavone moiety is a known pharmacophore against HIV-integrase. The aim of this work was to prepare a series of less cytotoxic protoflavone analogs and study their antiviral activity against HIV and EBV. Twenty-seven compounds, including 18 new derivatives, were prepared from apigenin through oxidative de-aromatization and subsequent continuous-flow hydrogenation, deuteration, and/or 4′-oxime formation. One compound was active against HIV at the micromolar range, and three compounds showed significant activity against the EBV lytic cycle at the medium-low nanomolar range. Among these derivatives, protoapigenone 1′-*O*-isopropyl ether (**6**) was identified as a promising lead that had a 73-times selectivity of antiviral over cytotoxic activity, which exceeds the selectivity of protoapigenone by 2.4-times. Our results open new opportunities for designing novel potent and safe anti-EBV agents that are based on the natural protoflavone moiety.

## 1. Introduction

**Figure 1:**
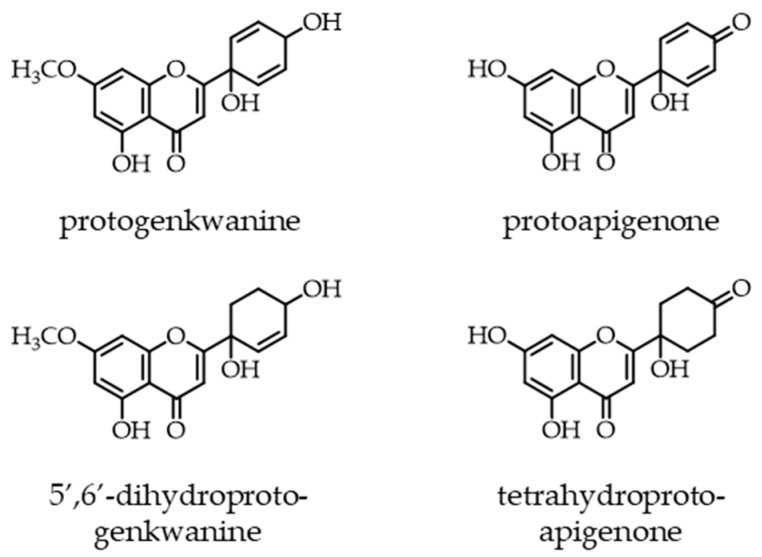
Examples of natural protoflavones with different moieties in their B-ring. A symmetric dienone structure, as in protoapigenone, is prerequisite of a strong cytotoxic activity.

Protoflavonoids represent a relatively rare, naturally occurring group of flavonoids, which possess a non-aromatic B-ring and a hydroxyl function at C-1′. This unique structural moiety can appear in various forms, it is most typically a symmetric dienone *p*-quinol that may be partially or fully saturated, as shown by the examples of some natural protoflavones illustrated in Figure 1 [1].

Many protoflavones are known for their potent anticancer activity and, until now, this is the bioactivity that is by far the most deeply investigated for this compound family. Protoflavones are known to be cytotoxic on a wide range of cancer cell lines in vitro, and some can selectively kill multi-drug resistant cancer cell lines that are adapted to chemotherapeutics [2,3]. Some protoflavones, e.g., protoapigenone, were also tested in vivo and found active against a variety of tumor xenograft models [1]. A particularly interesting pharmacodynamic property of protoapigenone is that it is a potent inhibitor of the ATR-mediated activation of checkpoint kinase 1 (Chk1) [4], a DNA damage response mechanism that is an emerging antitumor target in the focus of several currently ongoing clinical trials [5].

From the available structure-activity relationships that were obtained from synthetic [6] and semi-synthetic [7] analogs, it is known that (a) presence of a symmetric dienone with a non-substituted *p*-quinol B-ring is essential for a strong cytotoxic effect [8], (b) the presence of a longer non-branching (e.g., butyl) alkyl substituent at the 1′-OH can further enhance cytotoxicity against some cell lines, and (c) a branching 1′-*O*-alkyl substituent (e.g., isopropyl) strongly decreases cytotoxicity. It is also worth noting that a 1′-*O*-alkyl substitution results in a chemically much more stable B-ring when compared to the non-substituted *p*-quinol [7].

However, the possible use of protoflavonoids might exceed their antitumor potential. The non-aromatic B-ring containing an sp^3^ carbon at C-1^′^ makes the three-dimensional (3D) structure of these compounds very unique among flavonoids. This might lead to a versatile pharmacology that is hardly explored. Previously, we identified protoapigenone 1′-*O*-propargyl ether as the first non-planar flavonoid that can exert a potent inhibitory effect on xanthine oxidase, a pro-oxidant enzyme that is involved in many chronic diseases [9]. In addition to this, we found that protoapigenone exerts an antiviral effect against the Epstein–Barr Virus (EBV) in vitro through inhibiting the expression of EBV lytic proteins [10]. These results suggest that the chemical space of protoflavones has still much to offer in bioactivities other than antitumor effect, while cytotoxicity itself certainly represents a limitation for exploring these.

We recently reported a method for the highly selective saturation of the protoflavone B-ring by means of continuous flow hydrogenation under mild reaction conditions to overcome this limitation. This allowed for us to obtain the rare, naturally occurring tetrahydroprotoflavone moiety, while also providing an effective tool to eliminate the cytotoxicity of the derivatives [11].

The aim of our current work was to further explore the antiviral potential of protoflavonoids by preparing a series of less cytotoxic derivatives, and then test them against EBV and, while considering the antiretroviral activity of various other types of flavonoids [12,13,14], also against HIV.

## 2. Results and Discussion

### 2.1. Synthesis of B-Ring Modified Protoapigenone Analogs

First, we prepared the 1′-*O*-alkyl substituted protoapigenone derivatives from apigenin (**1**), following our previously described procedure [7]. Oxidative dearomatization of the B-ring of apigenin was achieved by a hypervalent iodine reagent, [bis(trifluoroacetoxy)iodo]benzene, and the solvent was 10% *v*/*v* of water or alcohol in acetonitrile depending on the substituent to be coupled at C-1′. This allowed for us to obtain compounds **2**–**8** as potential intermediates for further transformations, among which **6** was already a compound of interest for this study due to its very weak cytotoxicity when compared to the others.

**Scheme 1:**
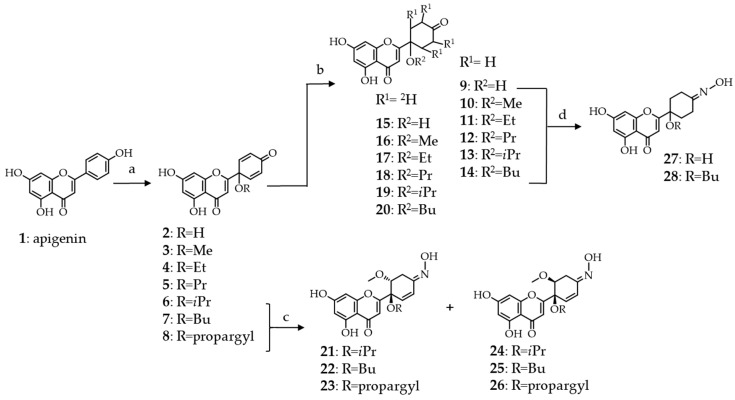
Semi-synthetic routes employed for the preparation of tetrahydro-, tetradeutero- or 4′-oxime analogs of protoapigenone and its 1′-*O*-alkyl ether derivatives. Oxime derivatives **21**–**28** were obtained as racemates, however, for simplicity only one enantiomer is shown. *Reaction conditions:* (**a**) CH_3_CN/ROH—9:1, PIFA (2 equiv.), 80°C, 1 h; (**b**) H-Cube^®^, **9**–**14**: H_2_, 5% Pd/C or **15**−**20**: D_2_, 5% Pd/BaSO_4_; (**c**) NH_2_OH·HCl (3 equiv.), MeOH, reflux, 24 h; and, (**d**) NH_2_OH·HCl (4 equiv.), MeOH, reflux, 3 h.

As detailed above, the function that is dominantly responsible for the cytotoxicity of protoflavones is the symmetric dienone moiety on the B-ring, conferring pro-oxidant and Micheal-acceptor properties to the compound. While considering this, we employed two different targeted synthetic strategies for eliminating cytotoxicity: (i) Saturation of the double bonds through hydrogenation or deuteration and (ii) substituting the 4′-oxo group with an oxime function. Scheme 1 shows synthetic routes.

The hydrogenation of protoflavonoids’ dienone moiety might result various products. Following our previously published procedure, the saturation of the protoflavone B-ring was achieved with high selectivity under mild reaction conditions while utilizing a modified H-cube^®^ continuous flow hydrogenation reactor [11]. Employing this device has several advantages when compared to traditional batch hydrogenation, i.e., the ease of handling of the explosive gas, precise control over the reaction conditions, instrumentally controlled gas pressure, sustainability, and safe applicability. During this process, hydrogen gas was in situ generated by an electrolytic cell fueled with high purity water, and forwarded to interact with the substrate’s solution. The mixture was then passed through a stainless-steel tube that was filled with the catalyst, where the triphasic reaction took place. During each transformation, the catalyst bed was placed in a thermostat to assure temperature control. Products of the reactions were collected into glass vials and subsequently purified by RP-HPLC to obtain tetrahydroprotoapigenone analogs **9**–**14** in high purity (Scheme 1).

Deuterium is applied in organic chemistry for several purposes. It is employed as a tracer in studies that aim to follow the reaction routes or as a reference for determining the influence of isotope effects on the development of a reaction during kinetic studies. Isotope labeling is a versatile technique that can be used many ways, including e.g., metabolomic studies, and the importance of deuterium in isotope labeling is increasing as new applications emerge [15,16].

Deuterium can also be of potential interest for discovery, since it can significantly influence bioactivity of a compound due to the so-called isotope effect [17,18]. Several methods are available for the synthetic preparation of deuterium containing compounds. The conventional batch synthesis of deuterated molecules suffers from similar drawbacks as hydrogenation, such as the lack of sustainability and the low purity and high price of D_2_ gas. Therefore, we used a previously established continuous-flow method in this current work [19,20,21], during which the required deuterium gas was in situ generated from high-purity D_2_O with H-Cube^®^. The reaction was performed in an aprotic solvent, ethyl acetate, to ensure that no hydrogen-deuterium exchange occurs during the transformation. For the same reason, we chose barium sulfate as the catalyst carrier, since activated charcoal might contain protic contaminations on its surface. In all other aspects of the synthesis, we followed the same procedure as in the case of hydrogenation, to obtain tetradeuteroprotoapigenone derivatives **15**–**20** (Scheme 1).

Several studies underline that the preparation of flavonoid oxime derivatives can be a simple yet effective synthetic option for enhancing certain biological effects of the parent compound, e.g., antimicrobial [22], antioxidant [23], and antitumor [24] properties. Compounds with different B-ring saturation and/or 1′-*O*-substituents (**6**–**9** and **14**) were selected for the preparation of 4′-oximes to further increase chemical and consequential pharmacological diversity (Scheme 1).

Our preliminary small-scale test reactions indicated that oxime formation of protoflavones is regioselective at the 4′-keto group, and under the synthetic conditions applied, we did not observe the formation of 4-oxime nor 4,4′-dioxime side products. On the other hand, the solvent appeared to play a crucial role in the possible outcome of the transformation. Pyridine, ethanol, and acetonitrile are probably among the most commonly used solvents in oxime synthesis. However, none of these worked in our case and, finally, we found methanol to be the best solvent for reacting protoflavones with hydroxylamine. The yields appeared to vary greatly, depending on the substrate’s concentration in the reaction solution, and we obtained the best yields with ca. 2–2.5 mg/mL concentrations; both lower and higher concentrations could significantly decrease the efficiency of the transformation. 

Regioselective transformation of the tetrahydroprotoflavone analogs **9** and **14** to their corresponding 4′-oximes **27** and **28** was straightforward. However, in the case of protoflavones **6**–**8**, each transformation was accompanied by a regioselective Michael addition of methanol at C-2′, forming a methoxy group and saturating one of the double bonds to yield 2′-methoxy-2′3′-dihydroprotoapigenone 4′-oxime derivatives **21**–**26**. The orientation of the methoxy group was apparently random, since the 1′,2′-*cis* and *trans* derivatives were formed in a ca. 1:1 ratio. When assigning the relative configuration of the 1′,2′-carbon atoms, we could not observe diagnostic NOESY cross-peaks between the 2′-methoxy and the 1′-*O*-alkyl substituents for any of these compounds. Therefore, *cis*-*trans* isomers were assigned based on the characteristic coupling constant patterns that were observed between H-2′ and H-3′a or H-3′b. To achieve this, structures were optimized by the MMFF94x force field in CCG-MOE, the H2′-C2′-C3′-H3′a, and H2′-C2′-C3′-H3′b dihedral angles were measured, and theoretical ^1^H-^1^H ^3^*J* coupling constants were calculated by means of the Bothner-By equation. The experimental coupling constants showed good agreement with the calculated values, unambiguously assigning compounds **21**–**23** as the 1′,2′-*trans*, and **24**–**26** as the 1′,2′-*cis* isomers. Nevertheless, while each compound was obtained as racemate, the newly formed oxime took a defined orientation in all of them. This suggests that the solvent addition took place to an intermediate of the reaction in a way that it determined the final orientation of the oxime. We considered compounds **27** and **28** as internal references, since the exact data of the Δδ syn-anti parameters were not available this way, which provided us with the chemical shifts for both neighbouring methylenes of the oxime. In these compounds, C-3′ (syn) and C-5′ (anti) gave ^13^C NMR chemical shifts at ca. 18.8 and 26.2 ppm, respectively. The ^13^C NMR signals of the 3′-CH_2_ moieties of compounds **21**–**26** appeared within the range of 22.7–23.7 ppm, which strongly suggests that the oxime is present in *E*-orientation in each of these compounds while also taking the effect of the electron-rich neighbouring OCH_3_ group into account.

### 2.2. Biological Activities

#### 2.2.1. Antiretroviral Activity

Compounds **2**, **6**–**14**, and **21**–**28** were tested against HIV-1 while using a pseudotype virus assay allowing only one cycle of viral replication and being more sensitive for compounds targeting the early steps of HIV replication, such as reverse transcription and integration [25]. Tetrahydroprotoapigenone (**9**) was found to inhibit viral infection by ca. 50% at the non-cytotoxic concentration of 100 µM (see Supplementary Materials, Table S1). While this represents a rather weak activity, it might still be of interest, since this compound was not cytotoxic on the host cells at as high as 500 µM concentration.

#### 2.2.2. Antiviral Activity against EBV

**Figure 2:**
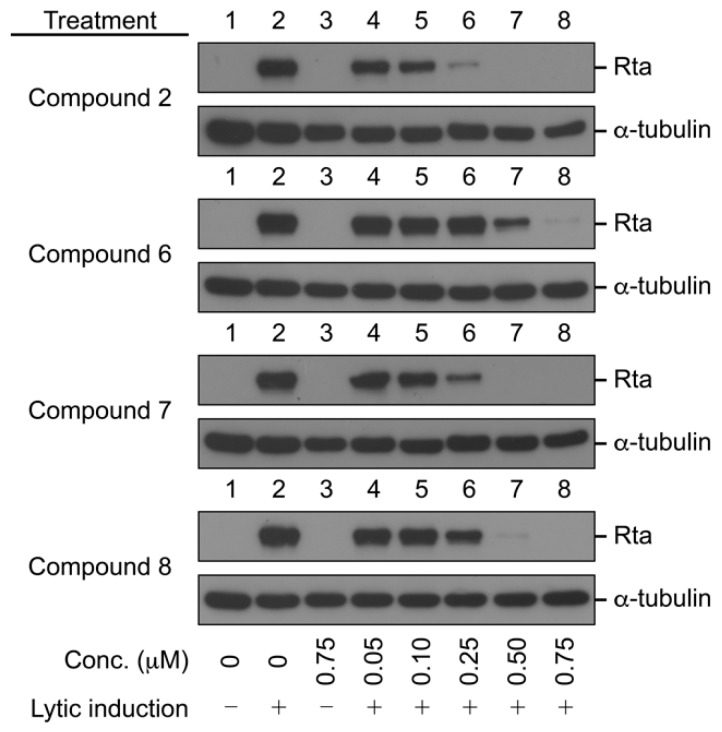
Inhibition of the expression of Epstein–Barr Virus (EBV) Rta by protoapigenone (**2**) and its analogs **6**–**8**. The cells were treated with the compounds at the time of lytic induction with SB and TPA. Cell lysates were harvested at 24 h after lytic induction. Proteins in the lysate were detected by immunoblotting using anti-Rta and anti-α-tubulin antibodies. Calculated IC_50_ values for **2**, **6**, **7,** and **8** were 0.127, 0.467, 0.208, and 0.285 µM, respectively.

Compounds **2** and **6**–**28** were tested for their activity on the EBV lytic cycle. To assess this, the P3HR1 cells were treated with sodium butyrate and TPA for 24 h. As expected, lytic protein Rta was expressed after lytic induction. As a first screening, the cells were treated with protoapigenone (**2**), used as a positive control, or compounds **6**–**28** at the induction of lytic cycle for 24 h. The concentration selected for screening was 0.25 µM, where protoapigenone (**2**) was previously found to exert a strong activity [10]. The results indicated that three of the analogs, **6**, **7**, and **8** can inhibit EBV lytic cycle at this concentration. These compounds were then tested for the dose dependency of their effect on the expression of Rta; Figure 2 shows the results.

**Figure 3:**
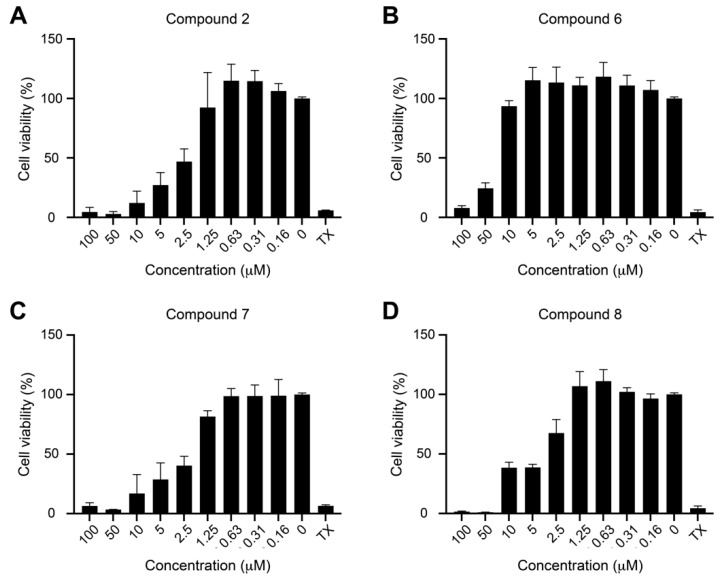
Cytotoxicity of protoapigenone (**A**) and its analogs (**B**–**D**) to P3HR1 cells. Cells were cultured for 24 h in a medium containing protoapigenone (**2**) or compounds **6**–**8**. Cell viability was evaluated by using MTT assay. Cells treated with 1% Triton X-100 (TX) were used as a positive control. The experiment was performed twice, and each sample involved in the experiment was prepared in duplicate. Error bars represent SD. Calculated IC_50_ values for compounds **2**, **6**, **7**, and **8** were 3.86, 34.12, 2.04, and 4.93 µM, respectively.

We found that, similarly to protoapigenone (**2**), compounds **6**, **7,** and **8** also caused a marked reduction in the Rta levels at 0.50, 0.25, and 0.50 µM, respectively, and the IC_50_ values were calculated as 0.467, 0.208, and 0.285 µM, respectively. The positive control protoapigenone (**2**) acted with an IC_50_ value of 0.127 µM, which was in good agreement with our previous findings [10]. Subsequently, we conducted MTT assay to evaluate the cytotoxicity of these compounds to P3HR1 cells; Figure 3 shows the results.

Compounds **7** and **8** were similarly active as protoapigenone, as expected from our previously published structure-activity relationship (SAR) study on the effect of 1′-*O*-alkyl substituents on the cytotoxic activity of protoflavones [7] (**2**), and the isopropyl-ether derivative **6** was much weaker in this regard. Selectivity of the anti-EBV vs. cytotoxic effect of these compounds, expressed as a ratio of the corresponding IC_50_ values, could therefore be calculated, as follows: **2** (30.1), **6** (73.0), **7** (9.80), and **8** (17.3). This means that compound **6** demonstrated a relevant, ca. 2.6-times increase in selectivity when compared to protoapigenone. It needs to be stressed that this selectivity increase is due to the much weaker cytotoxic activity of compound **6** as compared to protoapigenone, and it does not refer to a stronger anti-EBV activity. While this successfully fulfills our objectives that were set for this study, it might be worthy to further explore SAR regarding the role of the 1′-*O*-alkyl chain in the future. For example, when considering that the 1′-*O*-butyl derivative compound **7** exerted an over two-fold stronger anti-EBV activity than the 1′-*O*-isopropyl compound **6**, it might be a relevant strategy to study further analogs with a longer branching side-chain coupled at C-1′, such as *i*-butyl, *i*-pentyl, etc.

It is also worth noting that it was also found to be possible to shift the bioactivity profile of protoflavones to the other direction, i.e., increasing cytotoxicity and decreasing anti-EBV activity, by appropriate modifications of the B-ring. As compared with protoapigenone (**2**), compound **7** was less effective against EBV and more cytotoxic, i.e., ca. three-times less selective in this regard. Therefore, the SAR of protoflavonoids might be explored towards various therapeutic targets, and this is certainly a need to evaluate their real value concerning drug discovery.

## 3. Materials and Methods

### 3.1. Synthesis and Chromatographic Purification

Reagents were purchased from Sigma (Merck KGaA, Darmstadt, Germany) and the solvents were obtained from Macron Fine Chemicals (Avantor Performance Materials, Center Valley, Pennsylvania, USA). The reactions were monitored by TLC on Kieselgel 60F_254_ silica plates purchased from Merck (Merck KGaA, Darmstadt, Germany), and characteristic spots of compounds were examined under UV illumination at 254 and 366 nm. Chromatographic purification of components was carried out in one or two steps, depending on the complexity of protoflavone product mixtures. For flash chromatography, a CombiFlash^®^ Rf+ Lumen apparatus (TELEDYNE Isco, Lincoln, NE, USA) was utilized that was equipped with ELS and diode array detectors. Components were separated on commercially available RediSep NP-silica flash columns (TELEDYNE Isco, Lincoln, NE, USA) or manually filled polyamide columns. The mobile phase eluents consisted of mixtures of *n*-hexane—ethyl acetate or dichloromethane—methanol, modified according to the polarity of the analytes. To determine the composition of sample mixtures or to evaluate the purity of compounds, RP-HPLC analysis was performed on a Kinetex XB-C18 250 × 4.6 mm, 5 µm or a Kinetex Biphenyl 250 × 4.6 mm, 5 µm column (Phenomenex Inc., Torrance, CA, USA) at 1 mL/min. flow rate, while using a dual pump (PU-2080) Jasco HPLC instrument (Jasco International Co. Ltd., Hachioji, Tokyo, Japan) that was equipped with an MD-2010 Plus PDA detector to collect data in a detection range of 210–400 nm. For preparative purposes, an Armen Spot Prep II integrated HPLC purification system (Gilson, Middleton, WI, USA) with dual-wavelength detection was applied, operating at 230 and 300 nm. Preparative separations were performed on the corresponding Kinetex XB-C18 or Biphenyl 250 × 21.2 mm, 5 µm columns with adequately chosen eluents of acetonitrile–water, and the flow rates were 15 mL/min.

#### 3.1.1. Preparation of Protoapigenone Derivatives **2**–**8**

Protoapigenone and its 1′-*O*-alkyl ether analogs were synthesized while following our previously described procedures [7]. Briefly, apigenin (1 mg/mL) was dissolved in a 9:1 *v/v* ratio mixture of acetonitrile and H_2_O or the alcohol to be coupled at C-1′. Under stirring, two equivalents of [bis(trifluoroacetoxy)iodo]benzene was added slowly to the solution and the reaction was left to develop for 1 h at 80 °C. After completion, the solvent was evaporated on a rotary evaporator and the residue was purified by flash chromatography, allowing for obtaining the corresponding protoflavones in pure form.

#### 3.1.2. Preparation of Tetrahydroprotoapigenone Derivatives **9**–**14**

Selective hydrogenation of protoflavones was carried out based on our previous strategy [11] that relied on the application of an H-Cube^®^ continuous flow hydrogenation system (ThalesNano Inc., Budapest, Hungary). The applied catalyst cartridge was a stainless-steel column (internal dimensions: 50 mm × 4.6 mm) containing approximately 200 mg of 5% Pd/C catalyst. The cartridge was inserted into an external thermostat (Jetstream 2 plus, Jasco International Co. Ltd., Hachioji, Tokyo, Japan) set to 25 °C. A standard HPLC pump (Well-Chrom K-120, Knauer GmbH, Berlin, Germany) was used to provide continuous stream. In each case, the flow rate was set to 1 mL/min., and 40 bar pressure was applied in the system.

For each reaction, 20 mg of the corresponding protoflavone was dissolved/suspended in 20 mL of HPLC grade ethyl acetate and homogenized by sonication. The catalyst bed was washed with the solvent for 15 min. to ensure reproducibility between each reaction. Following transformations, preparative RP-HPLC was utilized for chromatographic purification.

#### 3.1.3. Preparation of Tetradeuteroprotoapigenone Derivatives **15**–**20**

The selective deuteration of the protoflavone B ring was performed similarly to that described for hydrogenation with only slight modifications. In this case, approximately 400 mg of 5% Pd/BaSO_4_ was used as a catalyst, and the reservoir of the instrument was filled up with high purity heavy water (D_2_O). The obtained compounds **15**–**20** were purified by preparative RP-HPLC.

#### 3.1.4. Preparation of Protoflavone 4′-oxime Derivatives **21**–**26**

An aliquot of 150 mg of compound **6**, **7**, or **8** was dissolved in methanol in a concentration of 2.5 mg/mL (50 mL). Following this, three equivalents of hydroxylamine hydrochloride were added to the solution, and the mixture was refluxed for 24 h at 70 °C. Silica gel (3–5 g) was added to the solution and the solvent was evaporated to prepare for dry loading separation. The dried residue was applied to flash chromatography, and products were pre-purified by a gradient separation program starting with *n*-hexane, and the ratio of ethyl acetate was gradually increasing 90%, *v/v*, in 15 min. Following this, cis- and trans-isomeric racemate pairs (**21** and **24**, **22** and **25**, **23**, and **26**, respectively) were separated by means of preparative RP-HPLC to obtain racemates **21**–**26**.

#### 3.1.5. Preparation of Tetrahydroprotoflavone 4′-oxime Derivatives **27**–**28**

An aliquot of 60 mg of tetrahydroprotoflavone **9** or **14** was dissolved in methanol in a concentration of 2.5 mg/mL (25 mL). Four equivalents of hydroxylamine hydrochloride were added to the mixture and the solution was stirred at 70 °C for 3 h. Following this, solvent was evaporated on a rotary evaporator and brine (40 mL) was added to the residue. Extraction was performed with EtOAc (3 × 40 mL), and the organic fractions were then combined and dried over Na_2_SO_4_. The drying agent was removed through filtration, and the solution was evaporated under reduced pressure. Eventually, preparative RP-HPLC was applied to obtain oxime derivatives **27** and **28**.

### 3.2. Structure Elucidation

NMR and MS techniques characterized the compounds. NMR spectra were recorded at 25 °C on a Bruker Avance DRX 400 MHz (Bruker Co., Billerica, MA, USA) or on a Bruker Avance NEO 500 MHz spectrometer that was equipped with a Prodigy BBO 5 mm CryoProbe with the use of TMS as an internal standard. Typically, 5–10 mg of the corresponding protoflavone was dissolved in DMSO-*d*_6_ and transferred to NMR tubes for recording spectra. The data report and spectra analysis were carried out with MestReNova v6.0.2-5475 software (Mestrelab Research S.L., Santiago de Compostela, Spain). ^1^H and ^13^C NMR chemical shifts are listed below; residual hydrogen signals of the B-rings of deuterated compounds **15**–**20** are marked with an asterisk. Mass spectra were recorded on an API 2000 triple quadrupole tandem mass spectrometer (AB SCIEX, Foster City, CA, USA) that was equipped with ESI ion source that was used in the negative ionization mode. ^1^H and ^13^C (JMOD) spectra of the new compounds are provided as Supplementary Materials, Figures S1–S36, and their mass spectra as Figures S37–S54.

Compound **10**: Pale yellow solid, Isolated yield: 25.2% (5.1 mg); RP-HPLC purity: 97%. ^1^H NMR (500 MHz, DMSO-*d*_6_): 12.65 (1H, s), 10.93 (1H, br s), 6.39 (1H, d, *J* = 1.9 Hz), 6.34 (1H, s), 6.20 (1H, d, *J* = 1.9 Hz), 3.22 (3H, s), 2.56 (2H, m), 2.35 (2H, m), 2.18–2.22 (4H, m). ^13^C NMR (125 MHz, DMSO-*d*_6_): 208.9, 181.7, 168.9, 164.5, 161.4, 157.8, 107.5, 103.9, 99.0, 94.1, 75.7, 51.0, 2 × 35.8, 2 × 30.8. ESI-MS (*m/z*): 303.4 [M-H]^−^.

Compound **11**: Pale yellow solid, Isolated yield: 70.5% (15.5 mg); RP-HPLC purity: 98%. ^1^H NMR (500 MHz, DMSO-*d*_6_): 12.66 (1H, s), 10.94 (1H, br s), 6.39 (1H, d, *J* = 1.9 Hz), 6.32 (1H, s), 6.20 (1H, d, *J* = 1.9 Hz), 3.40 (2H, q, *J* = 6.9 Hz), 2.55 (2H, m), 2.34 (2H, m), 2.16–2.22 (4H, m), 1.16 (3H, t, *J* = 6.9 Hz). ^13^C NMR (125 MHz, DMSO-*d*_6_): 209.0, 181.7, 169.6, 164.5, 161.4, 157.7, 107.1, 103.8, 99.0, 94.0, 75.5, 58.6, 2 × 35.9, 2 × 31.3, 15.5. ESI-MS (*m/z*): 317.7 [M-H]^−^.

Compound **12**: Pale brown solid, Isolated yield: 30.1% (6.1 mg); RP-HPLC purity: 99%. ^1^H NMR (500 MHz, DMSO-*d*_6_): 12.65 (1H, s), 10.97 (1H, br s), 6.38 (1H, d, *J* = 1.9 Hz), 6.32 (1H, s), 6.20 (1H, d, *J* = 1.9 Hz), 3.30 (2H, t, *J* = 6.4 Hz), 2.56 (2H, m), 2.36 (2H, m), 2.16–2.22 (4H, m), 1.55 (2H, m), 0.89 (3H, t, *J* = 7.4 Hz). ^13^C NMR (125 MHz, DMSO-*d*_6_): 209.0, 181.7, 169.3, 164.6, 161.4, 157.7, 107.3, 103.8, 99.0, 94.0, 75.2, 64.5, 2 × 35.9, 2 × 31.2, 22.8, 10.7. ESI-MS (*m/z*): 331.3 [M-H]^−^.

Compound **13**: Pale yellow solid, Isolated yield: 44% (8.9 mg); RP-HPLC purity: 99%. ^1^H NMR (500 MHz, DMSO-*d*_6_): 12.62 (1H, s), 10.99 (1H, br s), 6.42 (1H, s), 6.39 (1H, d, *J* = 1.9 Hz), 6.20 (1H, d, *J* = 1.9 Hz), 3.82 (1H, septet, *J* = 6.1 Hz), 2.55 (2H, m), 2.42 (2H, m), 2.13–2.21 (4H, m), 1.02 (6H, d, *J* = 6.1 Hz). ^13^C NMR (125 MHz, DMSO-*d*_6_): 209.2, 181.7, 169.0, 164.8, 161.4, 157.5, 108.0, 103.9, 99.1, 93.9, 75.0, 66.1, 2 × 36.1, 2 × 31.6, 2 × 24.0. ESI-MS (*m*/*z*): 331.4 [M-H]^−^.

Compound **15**: Pale yellow solid, Isolated yield: 63.1% (12.6 mg); RP-HPLC purity: 98.9%. ^1^H NMR (500 MHz, DMSO-*d*_6_): 12.73 (1H, s), 6.41 (1H, s), 6.38 (1H, d, *J* = 1.9 Hz), 6.18 (1H, d, *J* = 1.9 Hz), 6.08 (1H, br s), 2 × 2.26 * (d, *J* = 4.7 Hz), 2 × 2.17* (d, *J* = 4.7 Hz). ^13^C NMR (125 MHz, DMSO-*d*_6_): 209.6, 182.0, 174.3, 164.5, 161.4, 157.6, 104.7, 103.6, 98.9, 94.0, 70.2, 2 × 35.7, 2 × 33.7. ESI-MS (*m*/*z*): 293.5 [M-H]^−^.

Compound **16**: White solid, Isolated yield: 40.5% (8.2 mg); RP-HPLC purity: 95%. ^1^H NMR (500 MHz, DMSO-*d*_6_): 12.65 (1H, s), 10.96 (1H, br s), 6.39 (1H, d, *J* = 1.9 Hz), 6.34 (1H, s), 6.20 (1H, d, *J* = 1.9 Hz), 3.21 (3H, s), 2.52* (br d, *J* = 6.1 Hz) 2.33* (br d, *J* = 6.1 Hz), 2 × 2.18* (m). ^13^C NMR (125 MHz, DMSO-*d*_6_): 209.1, 181.7, 168.9, 164.6, 161.4, 157.8, 107.6, 103.8, 99.0, 94.1, 75.7, 51.0, 2 × 35.4, 2 × 30.4. ESI-MS (*m*/*z*): 307.4 [M-H]^−^.

Compound **17**: Pale pink solid, Isolated yield: 60.6% (12.1 mg); RP-HPLC purity: 96%. ^1^H NMR (500 MHz, DMSO-*d*_6_): 12.66 (1H, s), 10.94 (1H, br s), 6.39 (1H, d, *J* = 1.9 Hz), 6.32 (1H, s), 6.21 (1H, d, *J* = 1.9 Hz), 3.40 (2H, q, *J* = 6.9 Hz), 2.54* (br d, *J* = 6.0 Hz), 2.32* (br d, *J* = 6.0 Hz), 2 × 2.17* (m), 1.16 (3H, t, *J* = 6.9 Hz). ^13^C NMR (125 MHz, DMSO-*d*_6_): 209.1, 181.7, 169.5, 164.6, 161.4, 157.7, 107.1, 103.8, 99.0, 94.0, 75.4, 58.6, 2 × 35.5, 2 × 30.9, 15.5. ESI-MS (*m*/*z*): 321.3 [M-H]^−^.

Compound **18**: White solid, Isolated yield: 51.4% (10.4 mg); RP-HPLC purity: 98%. ^1^H NMR (500 MHz, DMSO-*d*_6_): 12.64 (1H, s), 10.97 (1H, br s), 6.38 (1H, d, *J* = 1.9 Hz), 6.32 (1H, s), 6.20 (1H, d, *J* = 1.9 Hz), 3.30 (2H, t, *J* = 6.3 Hz), 2.53* (br d, *J* = 6.1 Hz), 2.34* (br d, *J* = 6.1 Hz), 2 × 2.17* (m), 1.55 (2H, m), 0.88 (3H, t, *J* = 7.3 Hz). ^13^C NMR (125 MHz, DMSO-*d*_6_): 209.6, 182.1, 169.7, 165.1, 161.9, 158.2, 107.8, 104.3, 99.5, 94.5, 75.5, 64.9, 2 × 35.9, 2 × 31.3, 23.2, 11.1. ESI-MS (*m*/*z*): 335.5 [M-H]^−^.

Compound **19**: White solid, Isolated yield: 55.8% (11.3 mg); RP-HPLC purity: 99%. ^1^H NMR (500 MHz, DMSO-*d*_6_): 12.62 (1H, s), 11.01 (1H, br s), 6.42 (1H, s), 6.39 (1H, d, *J* = 1.9 Hz), 6.21 (1H, d, *J* = 1.9 Hz), 3.81 (1H, septet, *J* = 6.1 Hz), 2.53* (br d, *J* = 5.9 Hz), 2.39* (br d, *J* = 5.9 Hz), 2.17* (m), 2.13* (m), 1.01 (6H, d, *J* = 6.1 Hz). ^13^C NMR (125 MHz, DMSO-*d*_6_): 209.4, 181.7, 169.0, 164.8, 161.4, 157.5, 108.0, 103.8, 99.1, 93.9, 75.0, 66.0, 2 × 35.7, 2 × 31.2, 2 × 24.0. ESI-MS (*m*/*z*): 335.5 [M-H]^−^. 

Compound **20**: White solid, Isolated yield: 73.5% (14.7 mg); RP-HPLC purity: 99%. ^1^H NMR (500 MHz, DMSO-*d*_6_): 12.64 (1H, s), 10.98 (1H, br s), 6.37 (1H, d, *J* = 1.7 Hz), 6.32 (1H, s), 6.19 (1H, d, *J* = 1.7 Hz), 3.33 (2H, overlap with H_2_O signal, *J* = 5.7 Hz), 2.53* (br d, *J* = 6.1 Hz), 2.34* (br d, *J* = 6.1 Hz), 2 × 2.17 * (m), 1.52 (2H, m), 1.36 (2H, m), 0.85 (3H, t, *J* = 7.4 Hz). ^13^C NMR (125 MHz, DMSO-*d*_6_): 209.2, 181.6, 169.3, 164.7, 161.4, 157.7, 107.4, 103.8, 99.1, 94.0, 75.1, 62.5, 2 × 35.5, 31.6, 2 × 30.8, 18.9, 13.8. ESI-MS (*m*/*z*): 349.8 [M-H]^−^.

Racemate **21**: Pale brown solid; Isolated yield: 24.3% (41.67 mg); RP-HPLC purity at 241 nm: 99.5%. ^1^H NMR (500 MHz, DMSO-*d*_6_): 12.69 (1H, s), 11.45 (1H, s), 6.50 (1H, d, *J* = 10.1 Hz), 6.35 (1H, s), 6.34 (1H, d, *J* = 1.8 Hz), 6.32 (1H, d, *J* = 10.1 Hz), 6.20 (1H, d, *J* = 1.8 Hz), 3.91 (1H, septet, *J* = 6.1 Hz), 3.71 (1H, dd, *J* = 10.0 and 4.4 Hz), 3.18 (3H, s), 3.01 (1H, dd, *J* = 16.7 and 4.4 Hz), 2.43 (1H, dd, *J* = 16.7 and 10.0 Hz), 1.11 (3H, d, *J* = 6.1 Hz), 1.07 (3H, d, *J* = 6.1 Hz). ^13^C NMR (125 MHz, DMSO-*d*_6_): 181.5, 169.9, 164.6, 161.5, 157.6, 152.3, 130.1, 129.9, 108.4, 103.9, 99.1, 94.0, 79.0, 77.9, 67.6, 57.3, 24.03, 24.02, 23.6. ESI-MS (*m*/*z*): 374.7 [M-H]^−^.

Racemate **22**: Pale brown solid; Isolated yield: 27.8% (47.43 mg); RP-HPLC purity at 247 nm: 99.9%. ^1^H NMR (500 MHz, DMSO-*d*_6_): 12.68 (1H, s), 11.43 (1H, br s), 6.48 (1H, d, *J* = 10.0 Hz), 6.33 (1H, d, *J* = 2.0 Hz), 6.29 (1H, d, *J* = 10.0 Hz), 6.25 (1H, s), 6.21 (1H, d, *J* = 2.0 Hz), 3.76 (1H, dd, *J* = 10.1 and 4.7 Hz), 3.43 (2H, m), 3.18 (3H, s), 3.03 (1H, dd, *J* = 16.8 and 4.7 Hz), 2.45 (1H, dd, *J* = 16.8 and 10.2 Hz), 1.50 (2H, m), 1.32 (2H, m), 0.85 (3H, t, *J* = 7.4 Hz). ^13^C NMR (125 MHz, DMSO-*d*_6_): 181.4, 168.5, 164.5, 161.5, 157.8, 152.2, 130.1, 129.7, 108.2, 103.9, 99.1, 94.0, 78.6, 77.7, 64.1, 57.5, 31.8, 23.7, 18.8, 13.7. ESI-MS (*m*/*z*): 388.0 [M-H]^−^.

Racemate **23**: Pale brown solid; Isolated yield: 25.1% (43.11 mg); RP-HPLC purity at 248 nm: 99.8%. ^1^H NMR (500 MHz, DMSO-*d*_6_): 12.67 (1H, s), 11.52 (1H, s), 6.52 (1H, d, *J* = 10.0 Hz), 6.33 (1H, d, *J* = 1.9 Hz), 6.32 (1H, s), 6.29 (1H, d, *J* = 10.0 Hz), 6.21 (1H, d, *J* = 1.9 Hz), 4.27 (1H, dd, *J* = 15.7 and 2.3 Hz), 4.18 (1H, dd, *J* = 15.7 and 2.3 Hz), 3.80 (1H, dd, *J* = 10.2 and 4.7 Hz), 3.46 (1H, t, *J* = 2.3 Hz), 3.18 (3H, s), 3.07 (1H, dd, *J* = 16.7 and 4.7 Hz), 2.43 (1H, dd, *J* = 16.7 and 10.2 Hz). ^13^C NMR (125 MHz, DMSO-*d*_6_): 181.5, 167.8, 164.5, 161.5, 157.7, 152.1, 130.8, 128.7, 108.4, 104.0, 99.1, 94.1, 80.5, 78.33, 78.31, 77.5, 57.4, 53.3, 23.7. ESI-MS (*m*/*z*): 370.3 [M-H]^−^.

Racemate **24**: Pale brown solid; Isolated yield: 29.6% (50.76 mg); RP-HPLC purity at 246 nm: 97.3%. ^1^H NMR (500 MHz, DMSO-*d*_6_): 12.66 (1H, s), 11.38 (1H, s), 6.47 (3H, m, overlapping signals), 6.36 (1H, d, *J* = 1.9 Hz), 6.21 (1H, d, *J* = 1.9 Hz), 3.84–3.92 (2H, m, overlapping signals), 3.12 (1H, dd, *J* = 12.3 and 4.2 Hz), 3.10 (3H, s), 2.52 (1H, dd, *J* = 3.4 Hz; partially overlapped with DMSO signal), 1.04 (3H, d, *J* = 6.1 Hz), 0.97 (3H, d, *J* = 6.1 Hz). ^13^C NMR (125 MHz, DMSO-*d*_6_): 181.5, 168.7, 164.7, 161.5, 157.4, 151.1, 130.1, 127.3, 108.9, 103.9, 99.1, 93.8, 78.2, 76.6, 66.6, 56.9, 24.4, 23.8, 22.7. ESI-MS (*m*/*z*): 374.7 [M-H]^−^.

Racemate **25**: Pale brown solid; Isolated yield: 33.2% (56.64 mg); RP-HPLC purity at 247 nm: 99.7%. ^1^H NMR (500 MHz, DMSO-*d*_6_): 12.65 (1H, br s), 11.39 (1H, s), 10.92 (1H, br s), 6.48 (1H, d, *J* = 10.3 Hz), 6.42 (1H, s), 6.36 (1H, d, *J* = 10.3 Hz), 6.32 (1H, d, *J* = 2.1 Hz), 6.21 (1H, d, *J* = 2.1 Hz), 3.90 (1H, br t, *J* = 4.3 Hz), 3.45 (1H, m), 3.28 (1H, m), 3.15 (3H, s), 3.02 (1H, dd, *J* = 17.4 and 5.1 Hz), 2.61 (1H, dd, *J* = 17.4 and 3.9 Hz), 1.45 (2H, m), 1.30 (2H, m), 0.82 (3H, t, *J* = 7.4 Hz). ^13^C NMR (125 MHz, DMSO-*d*_6_): 181.5, 168.0, 164.5, 161.5, 157.5, 151.1, 130.1, 127.6, 108.7, 103.9, 99.1, 93.8, 78.0, 77.3, 62.8, 57.2, 31.6, 23.3, 18.7, 13.6. ESI-MS (*m*/*z*): 388.2 [M-H]^−^.

Racemate **26**: Pale brown solid; Isolated yield: 31.8% (54.62 mg); RP-HPLC purity at 248 nm: 97.3%. ^1^H NMR (500 MHz, DMSO-*d*_6_): 12.65 (1H, s), 11.48 (1H, s), 6.53 (1H, d, *J* = 10.3 Hz), 6.44 (1H, s), 6.40 (1H, d, *J* = 10.3 Hz), 6.34 (1H, d, *J* = 1.9 Hz), 6.20 (1H, d, *J* = 1.9 Hz), 4.21 (1H, dd, *J* = 15.8 and 2.3 Hz), 4.15 (1H, dd, *J* = 15.8 and 2.3 Hz), 3.93 (1H, dd, *J* = 4.6 and 3.6 Hz), 3.39 (1H, t, *J* = 2.3 Hz), 3.14 (3H, s), 3.04 (1H, dd, *J* = 17.4 and 4.6 Hz), 2.57 (1H, dd, *J* = 17.4 and 3.6 Hz). ^13^C NMR (125 MHz, DMSO-*d*_6_): 181.5, 167.1, 164.6, 161.5, 157.5, 151.0, 131.1, 126.0, 109.0, 104.0, 99.1, 93.9, 80.3, 77.9, 77.6, 77.4, 57.2, 52.1, 23.1. ESI-MS (*m*/*z*): 370.2 [M-H]^−^.

Racemate **27**: Pale white solid; Isolated yield: 48.3% (30.5 mg); RP-HPLC purity at 247 nm: 96.1%. ^1^H NMR (500 MHz, DMSO-*d*_6_): 12.75 (1H, s), 10.34 (1H, s), 6.37 (1H, d, *J* = 1.9 Hz), 6.36 (1H, s), 6.18 (1H, d, *J* = 1.9 Hz), 5.84 (1H, br s), 3.08 (1H, br d, *J* = 14.2 Hz), 2.45 (1H, td, *J* = 13.4 and 4.9 Hz), 2.21 (1H, m), 2.06 (1H, m), 1.98 (1H, td, *J* = 12.9 and 4.6 Hz), 1.80–1.89 (3H, m, overlapping signals). ^13^C NMR (125 MHz, DMSO-*d*_6_): 182.1, 174.9, 164.4, 161.4, 157.6, 155.4, 104.5, 103.6, 98.9, 94.0, 71.2, 34.6, 33.3, 26.3, 18.8. ESI-MS (*m*/*z*): 304.4 [M-H]^−^.

Racemate **28**: Pale white solid; Isolated yield: 42.1% (26.4 mg); RP-HPLC purity at 250 nm: 96.6%. ^1^H NMR (500 MHz, DMSO-*d*_6_): 12.66 (1H, s), 10.36 (1H, s), 6.38 (1H, d, *J* = 1.9 Hz), 6.27 (1H, s), 6.19 (1H, d, *J* = 1.9 Hz), 3.28 (2H, t, *J* = 6.1 Hz), 3.05 (1H, br d, *J* = 14.4 Hz), 2.33 (1H, td, *J* = 13.9 and 5.4 Hz), 2.16–2.23 (3H, m, overlapping signals), 1.98 (1H, td, *J* = 13.6 and 5.1 Hz), 1.87 (1H, td, *J* = 14.2 and 4.3 Hz), 1.78 (1H, td, *J* = 13.3 and 4.6 Hz), 1.49 (2H, m), 1.35 (2H, m), 0.84 (3H, t, *J* = 7.4 Hz). ^13^C NMR (125 MHz, DMSO-*d*_6_): 181.7, 170.0, 164.5, 161.4, 157.7, 155.0, 107.1, 103.8, 99.0, 94.0, 76.0, 62.2, 31.8, 31.6, 30.6, 26.2, 18.9, 18.8, 13.8. ESI-MS (*m*/*z*): 360.5 [M-H]-.

### 3.3. Cell Lines and Viruses

The U373-CD4-CCR5 cell line was obtained from the AIDS Research and Reagent Program, NIAID. Cells were cultured according to the protocol of the provider with 10% heat-inactivated fetal bovine serum (Lonza, Maastricht, The Netherlands) supplemented with 2 mM l-glutamine, 100 units/mL of penicillin, and 100 µg/mL streptomycin (Invitrogen, Merelbeke, Belgium). Pseudotyped viral particules bearing the ADA HIV Env were produced, as described previously [25].

EBV-positive BL cell line P3HR1 cells were cultured in RPMI 1640 medium [26,27]. Culture media were supplemented with 10% fetal calf serum. P3HR1 cells were treated with 3 mM sodium butyrate and 30 nM 12-*O*-tetradecanoylphobol-13-actetae (TPA) to induce the EBV lytic cycle [26,27].

### 3.4. Anti-HIV Testing

Antiviral activity was assessed while using a Pseudotype Virus Assay, allowing for one cycle of viral replication [25]. The U373-CD4-CCR5 cells were infected by pseudotyped virus pNL4.3ΔenvLuc-ADA 8 using spinoculation at 1200 g during 2 h in the presence of compounds and cultured for two consecutive days in the presence of the compounds. After 48 h, luciferase activity expressed as Relative Light Units was measured (Luciferase System kit, Promega, The Netherlands). Drug cytotoxicity was evaluated while using MTT (3-(4,5-dimethylthiazol-2-yl)-2,5-diphenyltetrazolium bromide, Sigma, Belgium) 48 h after incubation with the compounds by measuring A540 and A690 using a POLARstar Omega Plate Reader (BMG Lab Technologies, Belgium) after 48 h of incubation with the compounds. The value of OD540–OD690 was calculated.

### 3.5. Immunoblot Analysis of EBV Rta Protein

P3HR1 cells were lysed in mRIPA buffer (50 mM Tris-HCl, pH 7.8, 150 mM NaCl, 5 mM EDTA, 0.5% Triton X-100, 0.5% NP-40) [26,27]. Approximately 5% of the lysate was loaded to a gel and then separated by electrophoresis in 10% SDS-PAGE. The proteins in the gel were electroblotted onto a polyvinylidene difluoride (PVDF) blotting membrane while using a Hoefer transfer system. Rta was detected while using mouse anti-Rta monoclonal antibody (Argene, Verniolle, France). α-Tubulin was detected using mouse anti-α-tubulin monoclonal antibody (Abcam, Cambridge, UK). Primary antibodies were detected using horseradish peroxidase-conjugated secondary antibodies (Cell Signaling Technology, Danvers, MA, USA) and then visualized using SuperSignal West Pico Chemiluminescent substrate (Thermo Fisher Scientific, Waltham, MA, USA).

### 3.6. Cytotoxicity Assay

P3HR1 cells (5 × 10^4^) were seeded in wells of a 96-well plate and then cultured for 24 h in 100 µL of RPMI 1640 medium containing protoapigenone or its analogs. Subsequently, 1% Triton X-100 was added as a negative control. Cytotoxicity was determined by Cell Proliferation Kit I (Roche, Basel, Switzerland). An aliquot of 10 µL of the MTT labeling reagent was added to each well and the plate was incubated for 4 h at 37°C. Afterwards, 100 µL solubilization solution was added to each well to dissolve insoluble formazan and was incubated overnight. Optical density was measured at 595 nm and 650 nm.

## 4. Conclusions

As a result of this work, the chemical space of non-cytotoxic protoflavonoids was significantly extended through the preparation of derivatives without the symmetric dienone moiety, such as saturated (hydrogenated or deuterated) B-ring and/or 4′-oxime containing protoflavones. Compound **9** was found to exert a weak antiretroviral activity that, due to the extremely low cytotoxicity of this compound, might still be of interest. Further, three compounds, the isopropyl-, butyl-, and propargyl-ether derivatives of protoapigenone were identified to act as potent anti-EBV agents at similar, medium-low nanomolar concentration range as their parent compound **2**. Importantly, protoapigenone 1′-*O*-isopropyl ether demonstrated an order of magnitude lower cytotoxic effect when compared to protoapigenone, which makes compound **6** ca. 2.6-times more selective anti-EBV agent. Therefore, this compound can serve as a possible new lead for the development of safe and effective protoflavone derivatives against the lytic cycle of EBV.

Altogether, as a result of this work, it might also be concluded that targeted chemical modifications of the protoflavone B-ring to decrease cytotoxicity represent a valid strategy towards the discovery of new bioactive compounds against pathologies other than cancer.

## Supplementary Materials

Supplementary materials can be found at https://www.mdpi.com/1422-0067/20/24/6269/s1.
