# Supplementary material for: Less Cytotoxic Protoflavones as Antiviral Agents: Protoapigenone 1′-O-isopropyl ether Shows Improved Selectivity Against the Epstein–Barr Virus Lytic Cycle
Source: Int J Mol Sci. 2019 Dec 12;20(24):6269. doi: 10.3390/ijms20246269 (PMC6940897; doi:10.3390/ijms20246269)
Supplement: Supplementary file 1 [file ijms-20-06269-s001.pdf]

Less cytotoxic protoflavones as antiviral agents: protoapigenone 1′-*O*-isopropyl ether shows improved selectivity against the Epstein-Barr virus lytic cycle

Supporting information

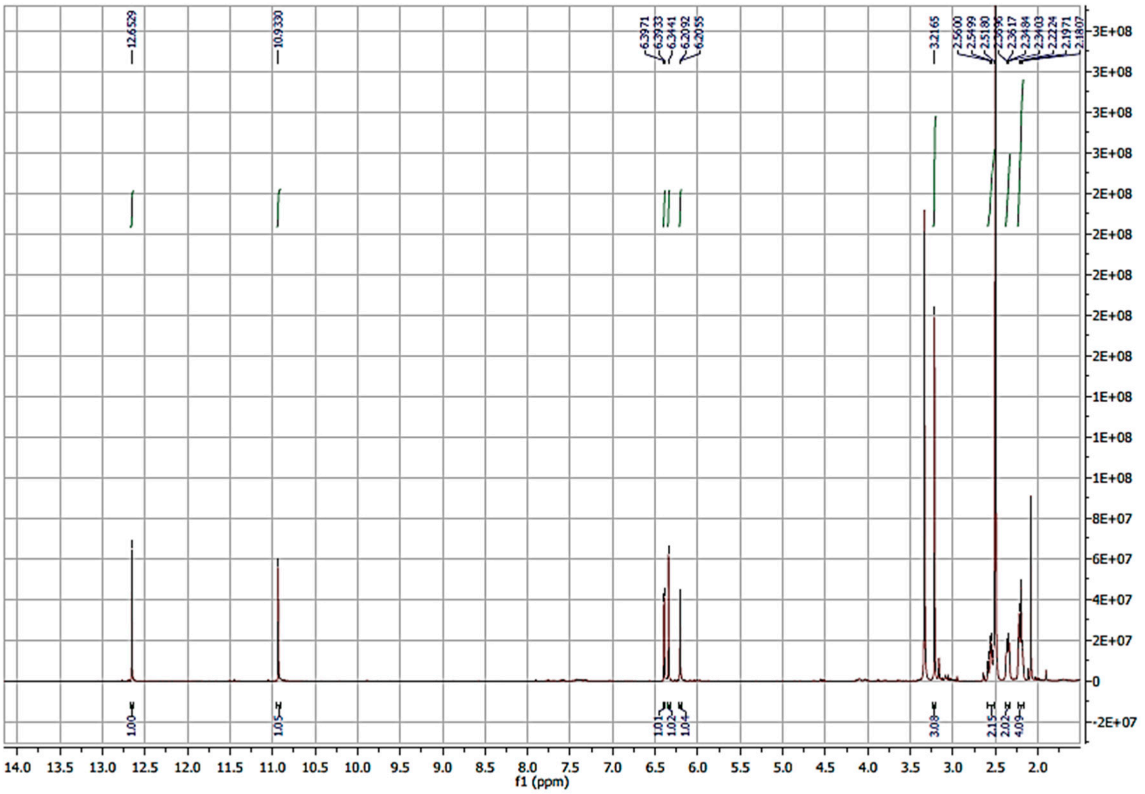

Figure S1. ^{1}H NMR (500 MHz, DMSO-*d*$_6$) spectrum of compound **10**.

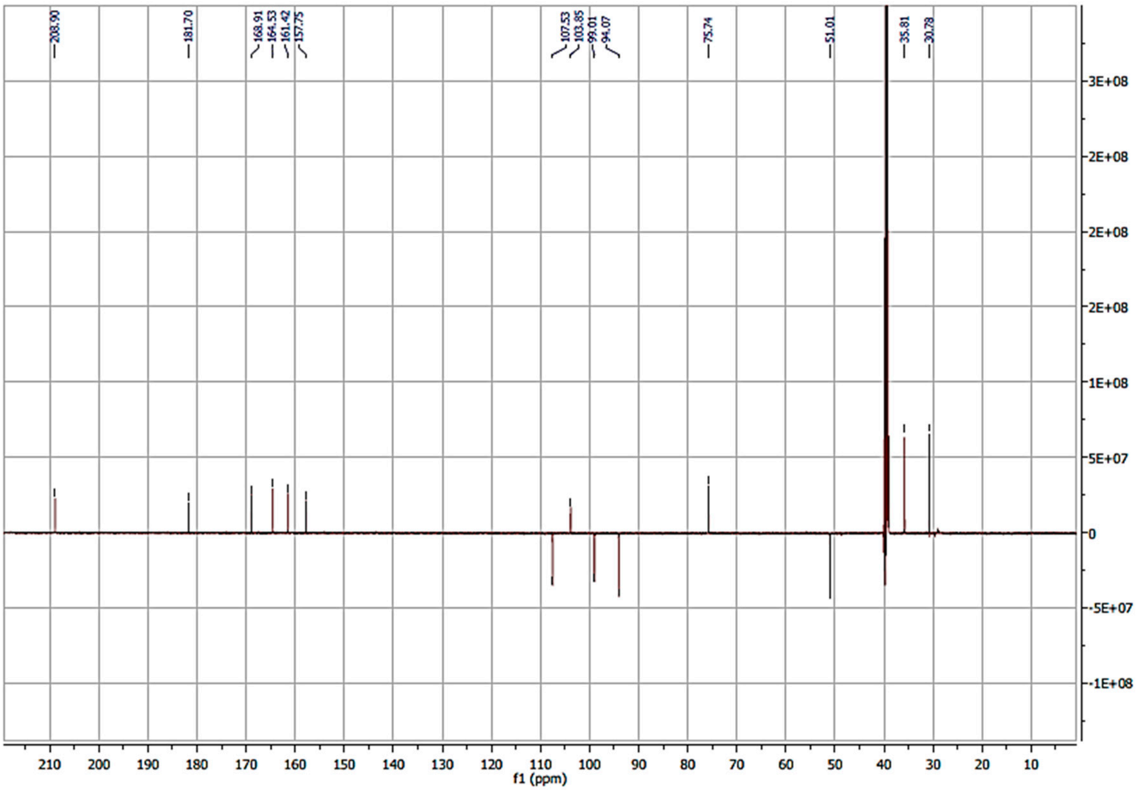

Figure S2. JMOD (125 MHz, DMSO-*d*$_6$) spectrum of compound **10**.

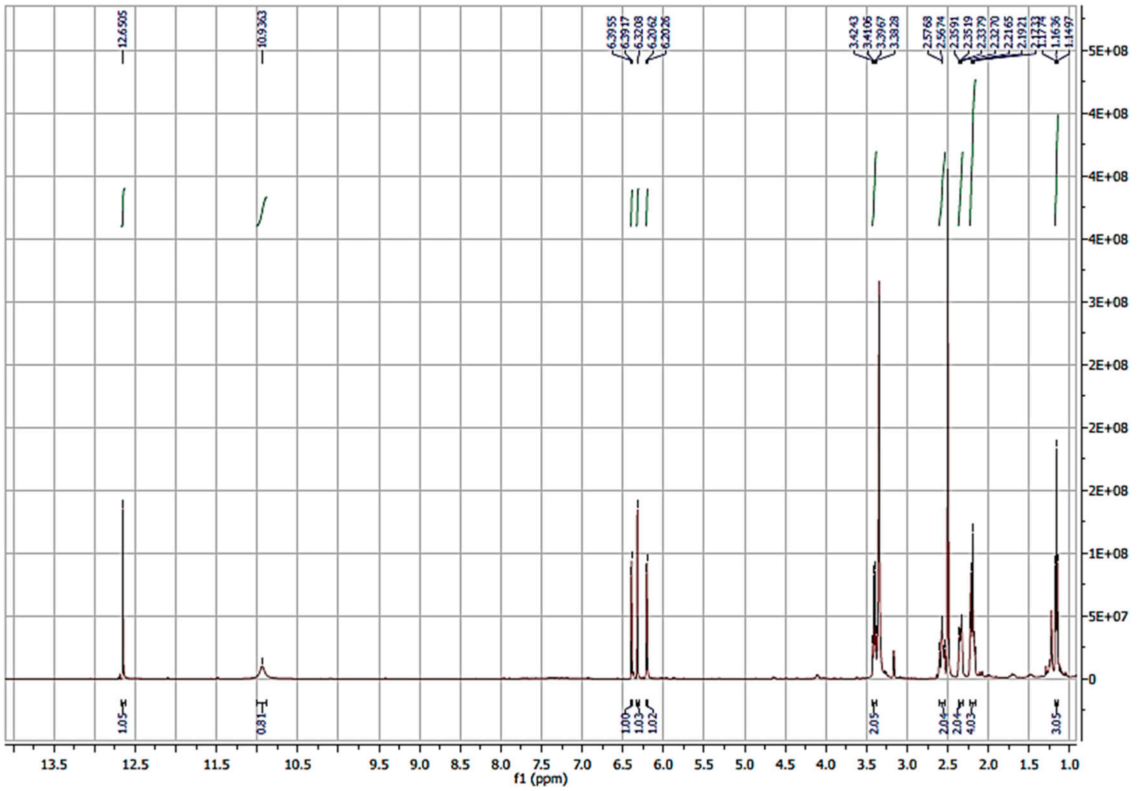

Figure S3. ^{1}H NMR (500 MHz, DMSO-d_6) spectrum of compound **11**.

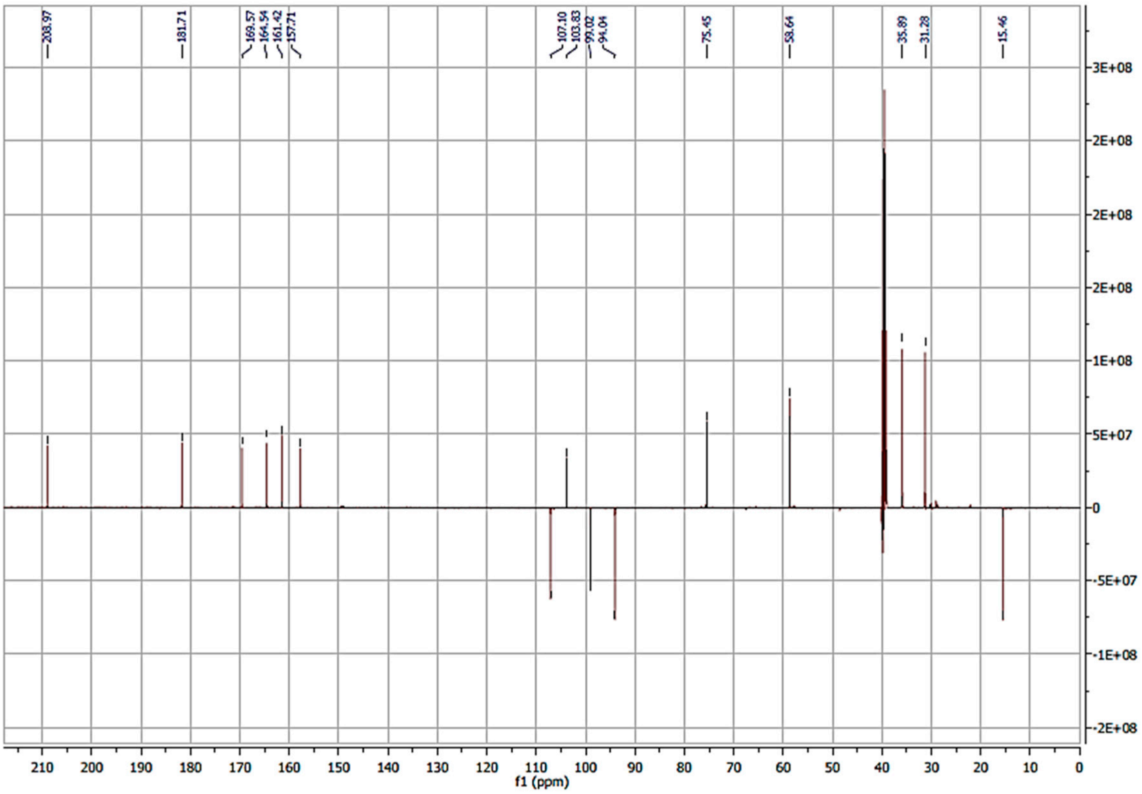

Figure S4. JMOD (125 MHz, DMSO-d_6) spectrum of compound **11**.

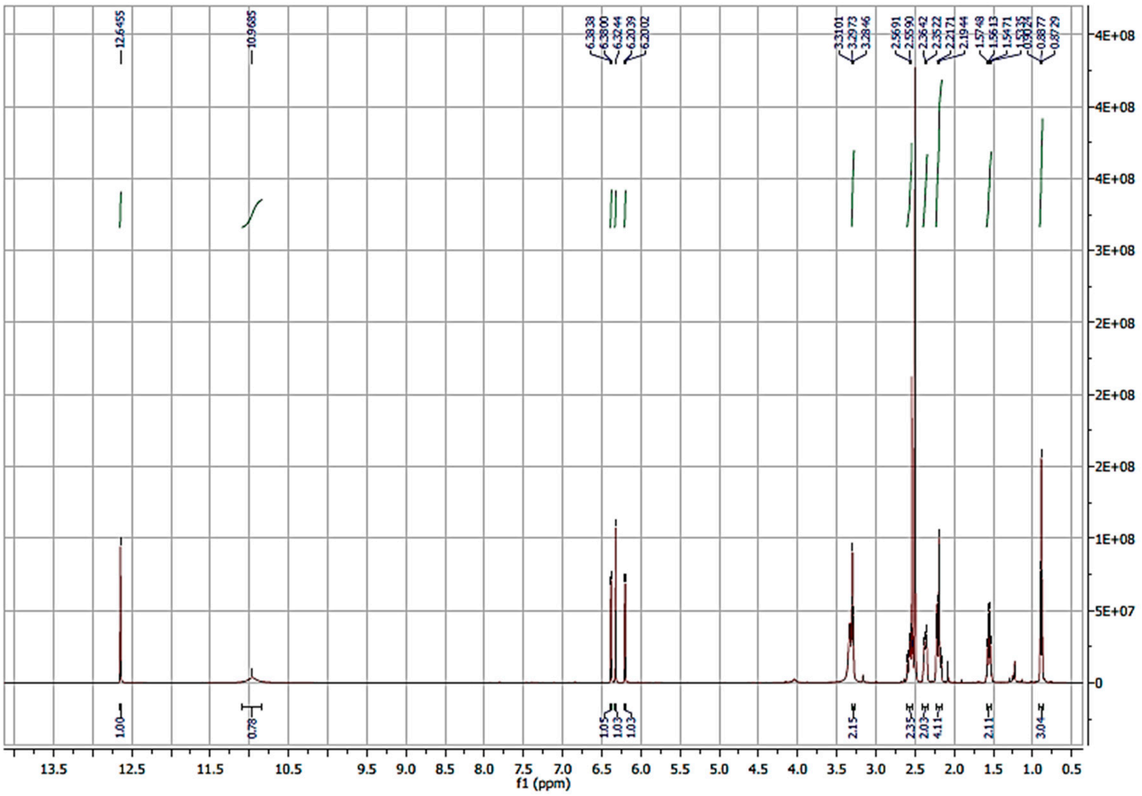

Figure S5. ^{1}H NMR (500 MHz, DMSO-d_6) spectrum of compound **12**.

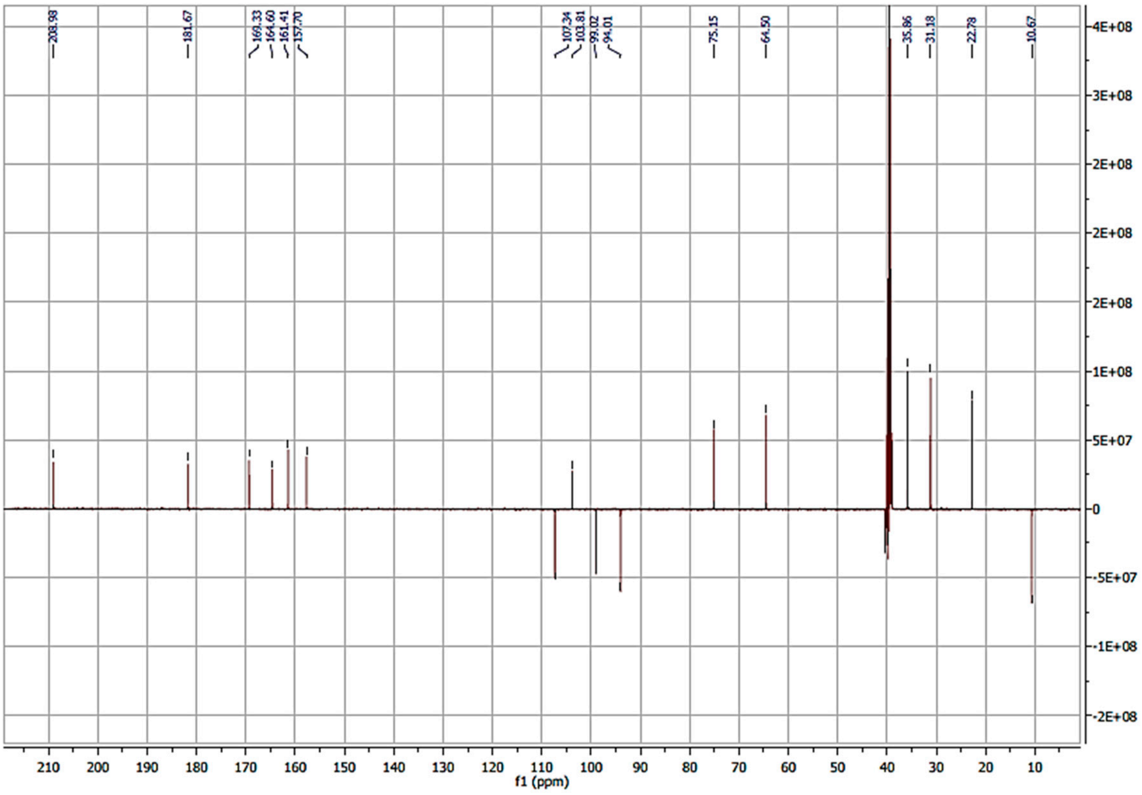

Figure S6. JMOD (125 MHz, DMSO-d_6) spectrum of compound **12**.

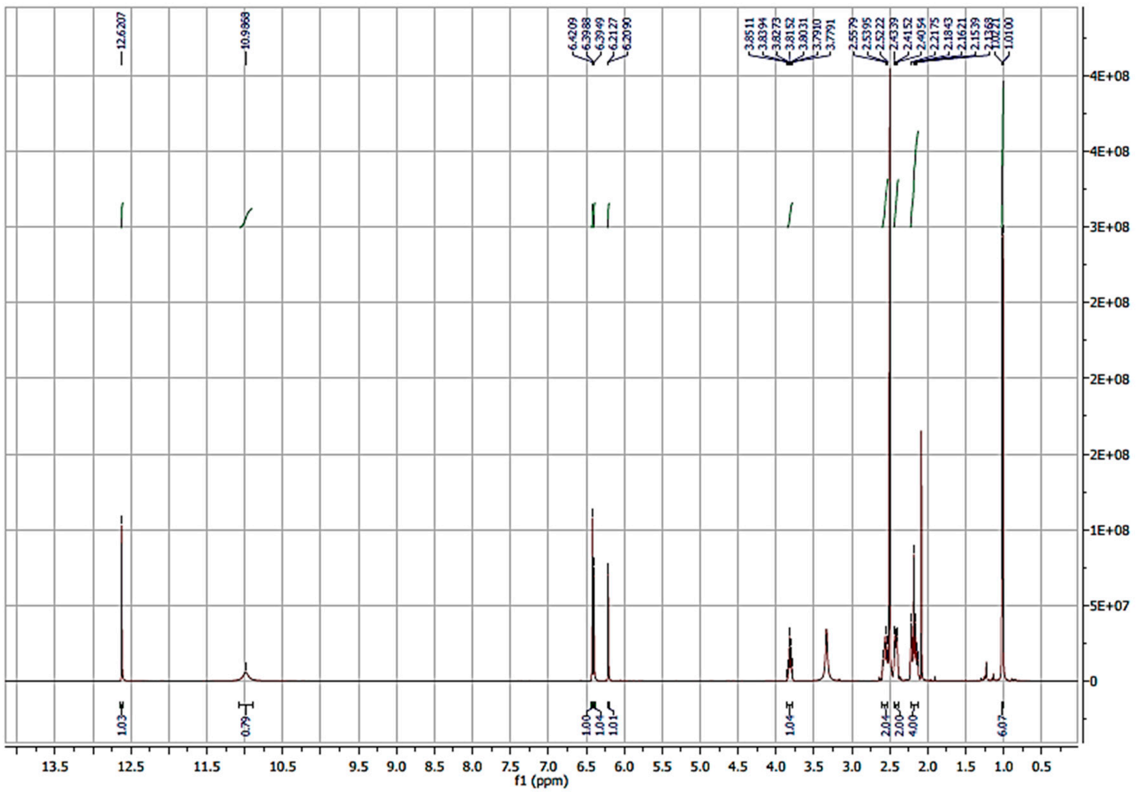

Figure S7. ^{1}H NMR (500 MHz, DMSO-*d*6) spectrum of compound **13**.

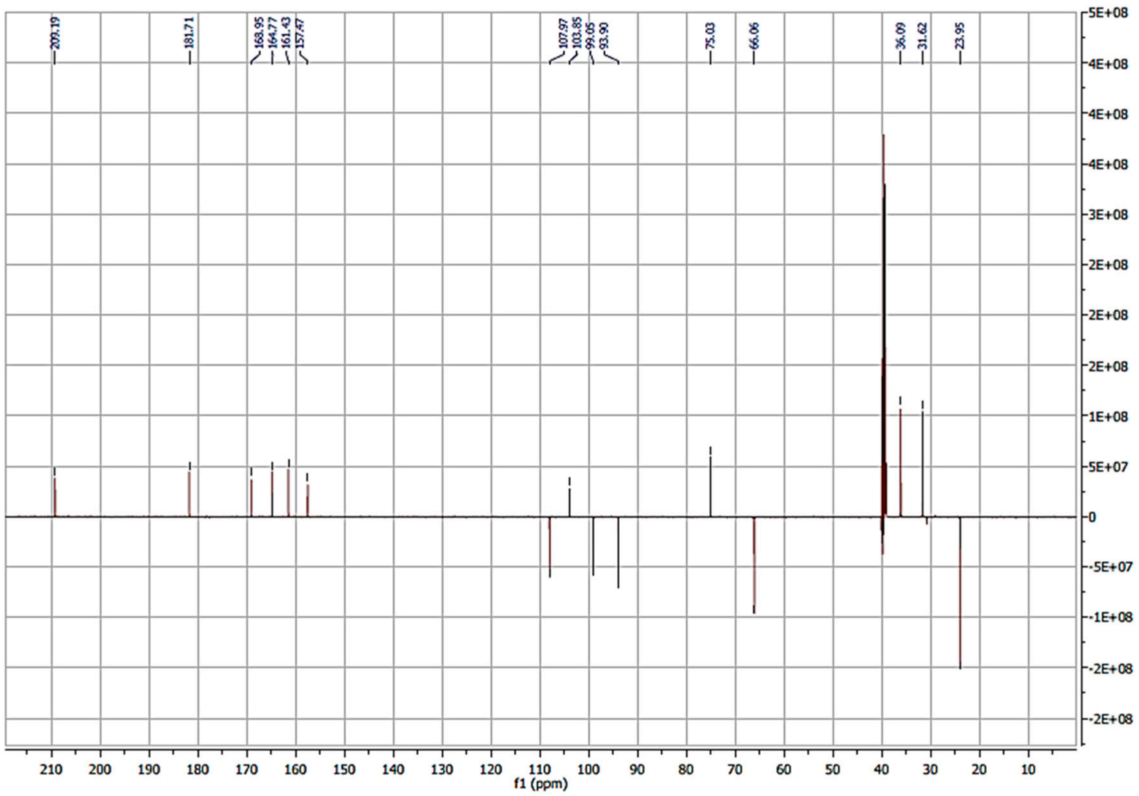

Figure S8. JMOD (125 MHz, DMSO-*d*6) spectrum of compound **13**.

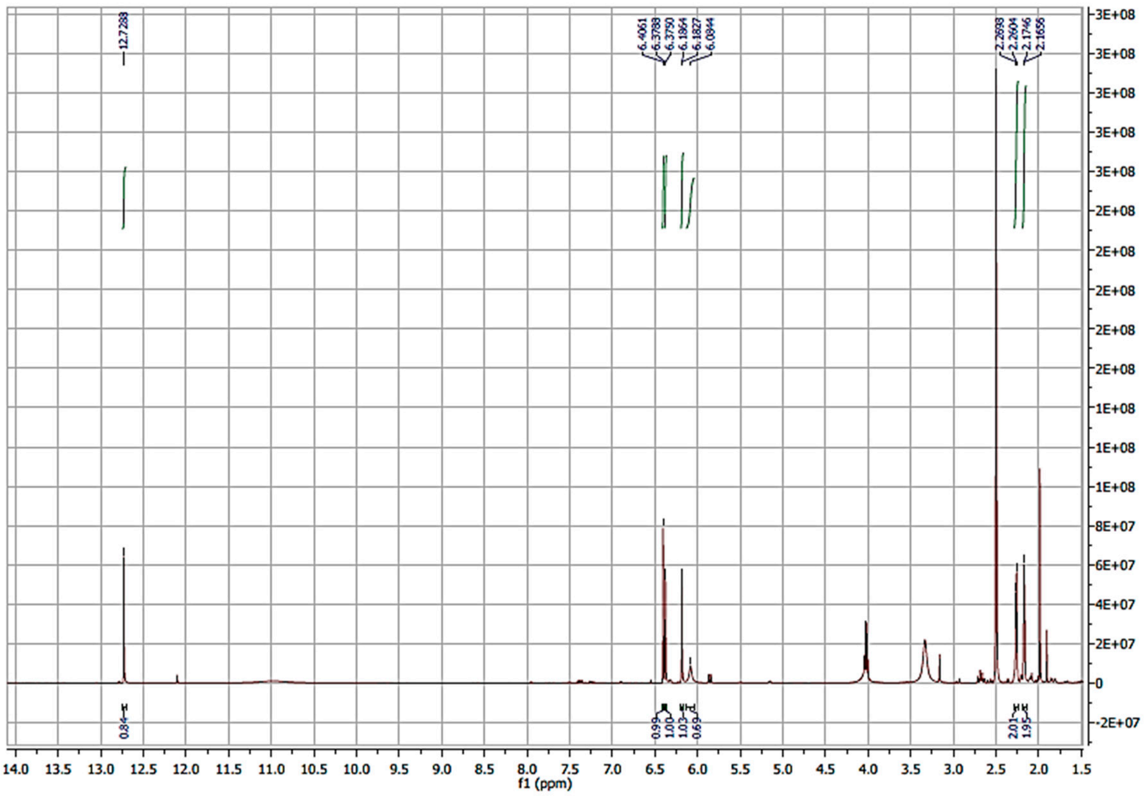

Figure S9. ^{1}H NMR (500 MHz, DMSO-d_6) spectrum of compound **15**.

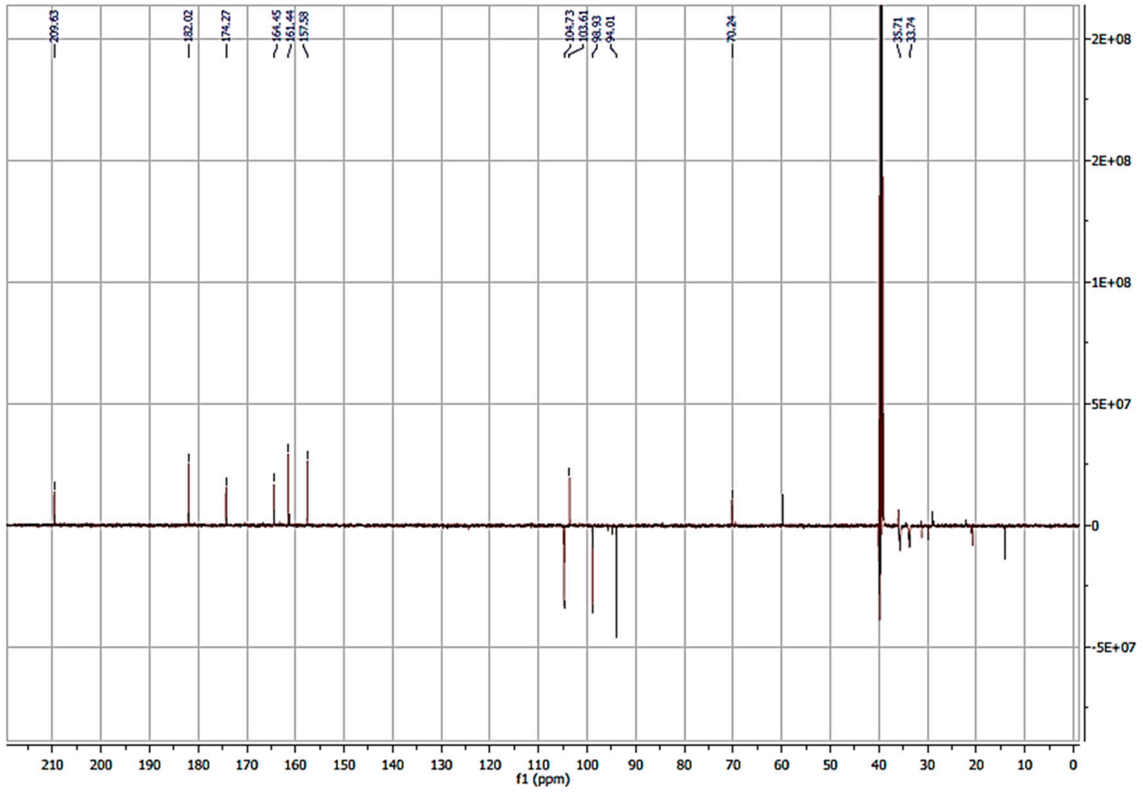

Figure S10. JMOD (125 MHz, DMSO-d_6) spectrum of compound **15**.

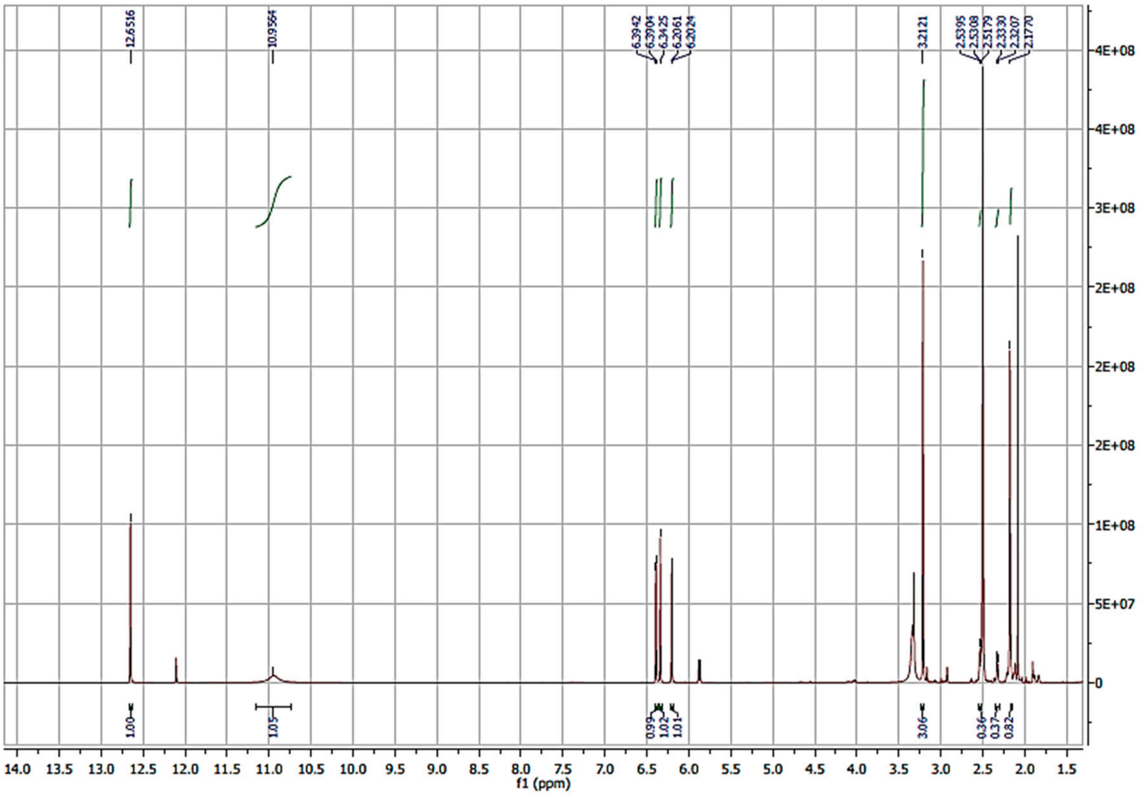

Figure S11. ^{1}H NMR (500 MHz, DMSO-d_6) spectrum of compound **16**.

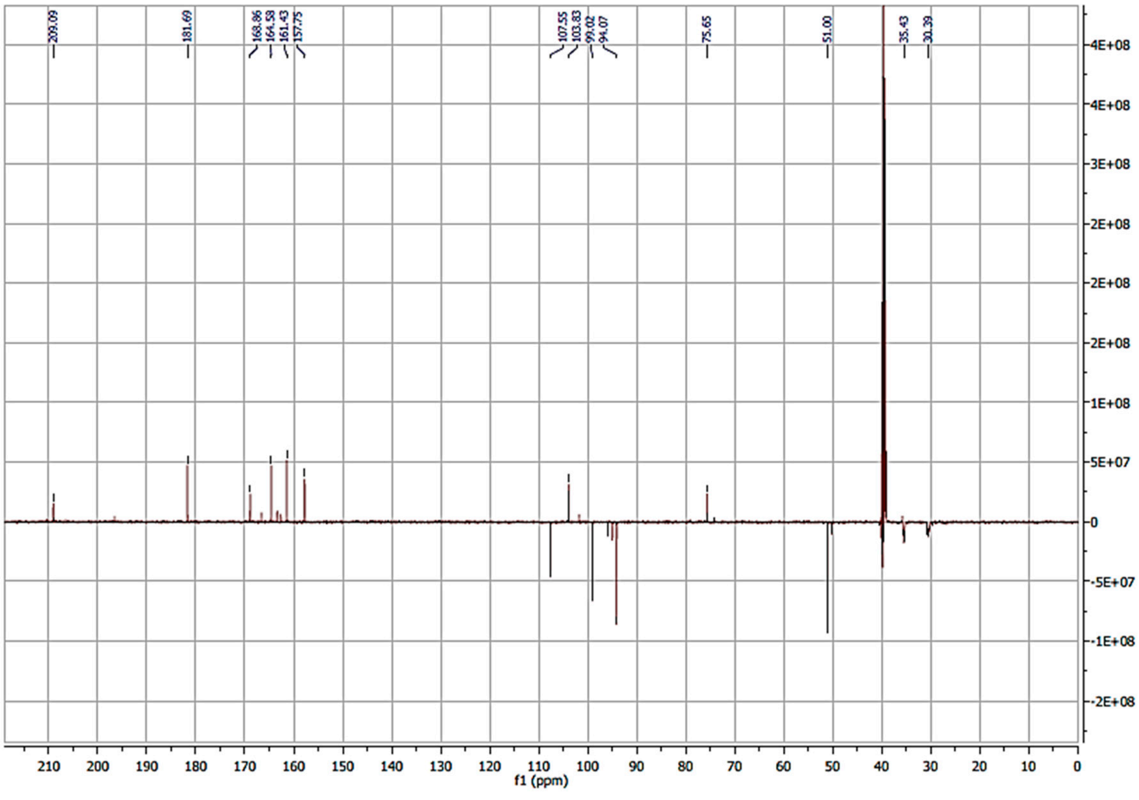

Figure S12. JMOD (125 MHz, DMSO-d_6) spectrum of compound **16**.

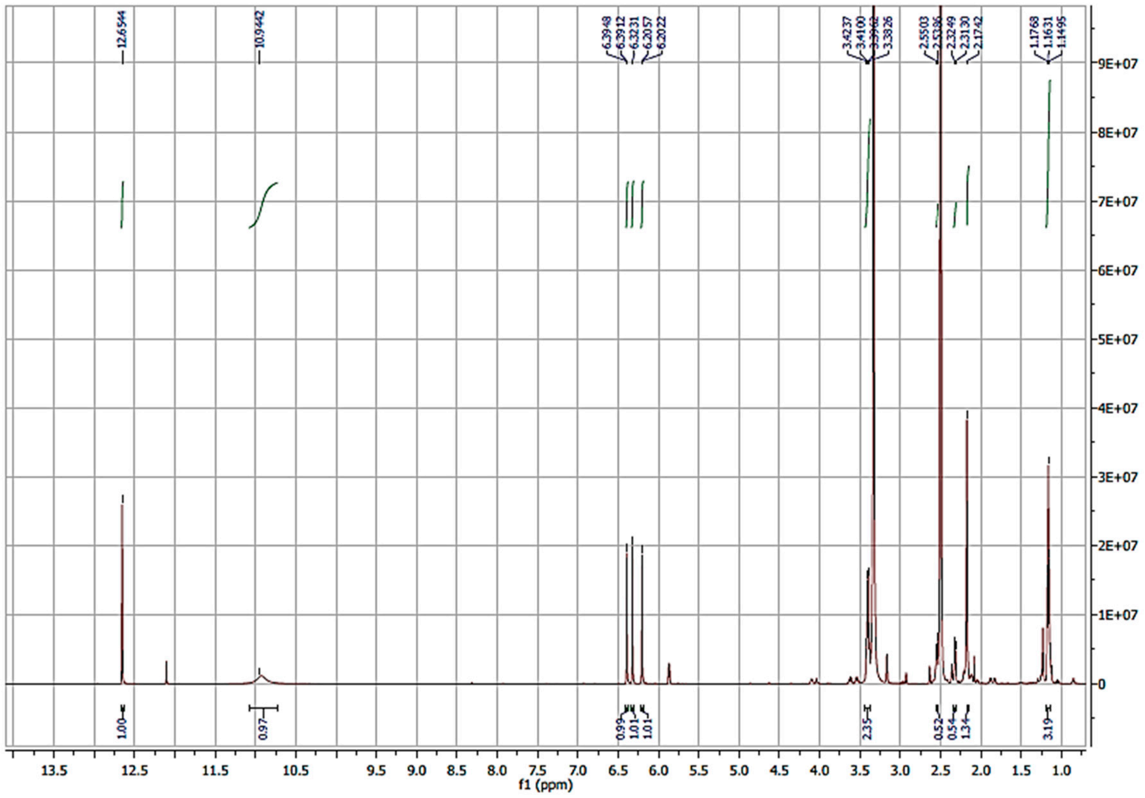

Figure S13. ^{1}H NMR (500 MHz, DMSO-d_6) spectrum of compound **17**.

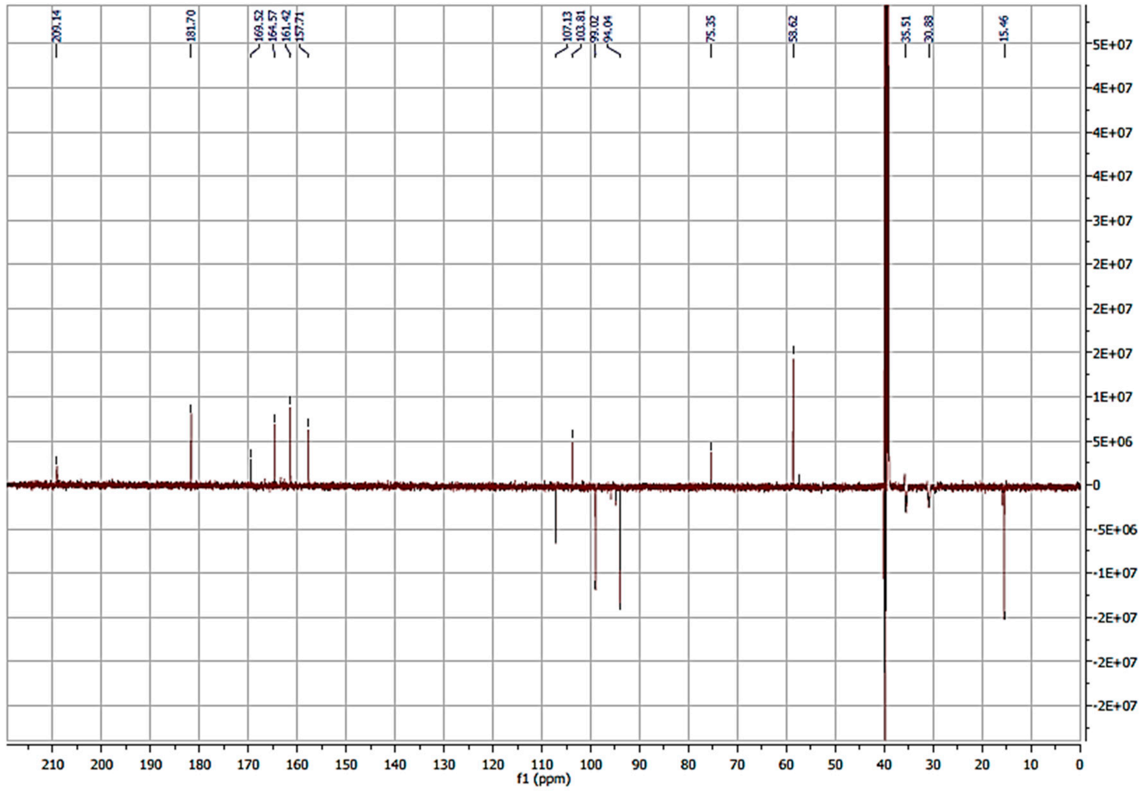

Figure S14. JMOD (125 MHz, DMSO-d_6) spectrum of compound **17**.

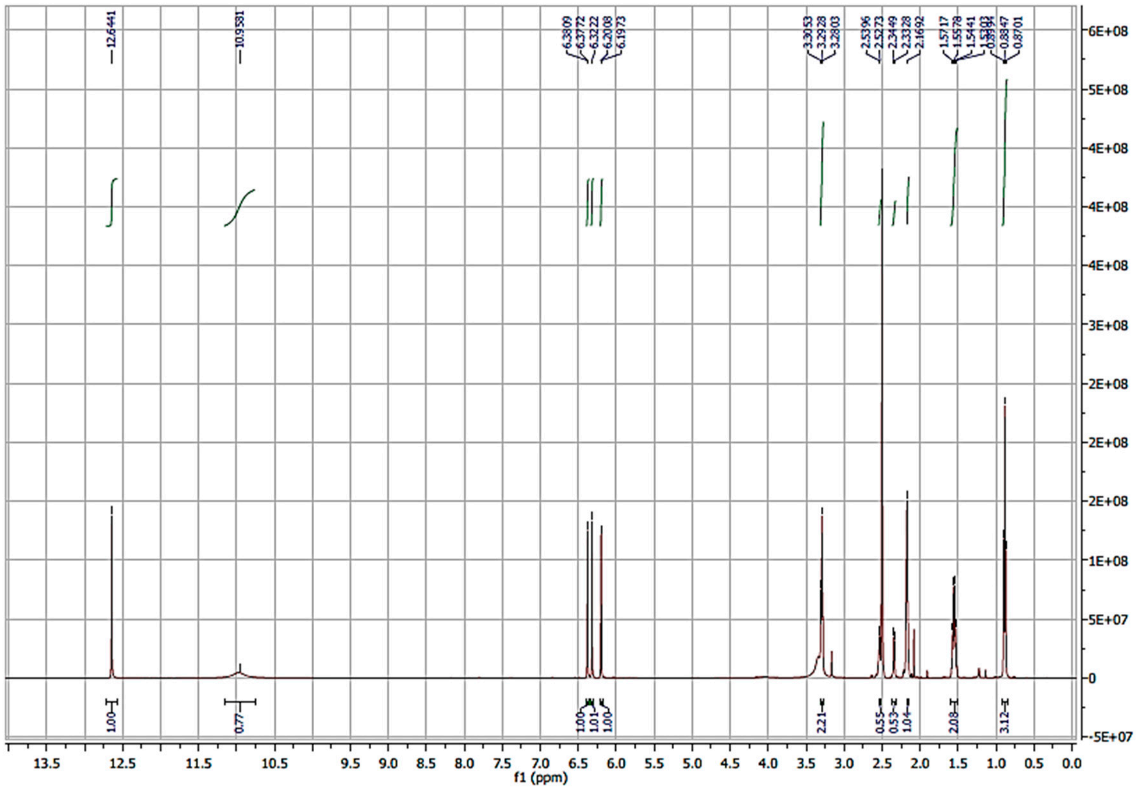

Figure S15. ^{1}H NMR (500 MHz, DMSO-d_6) spectrum of compound **18**.

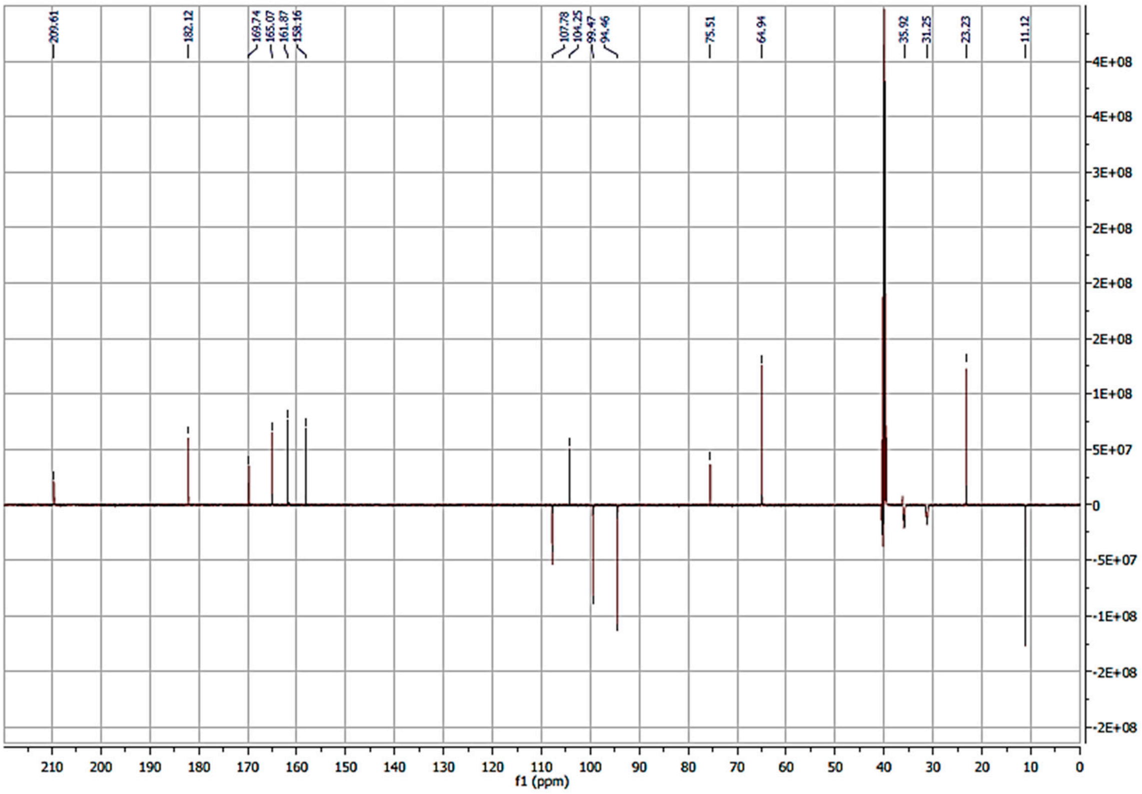

Figure S16. JMOD (125 MHz, DMSO-d_6) spectrum of compound **18**.

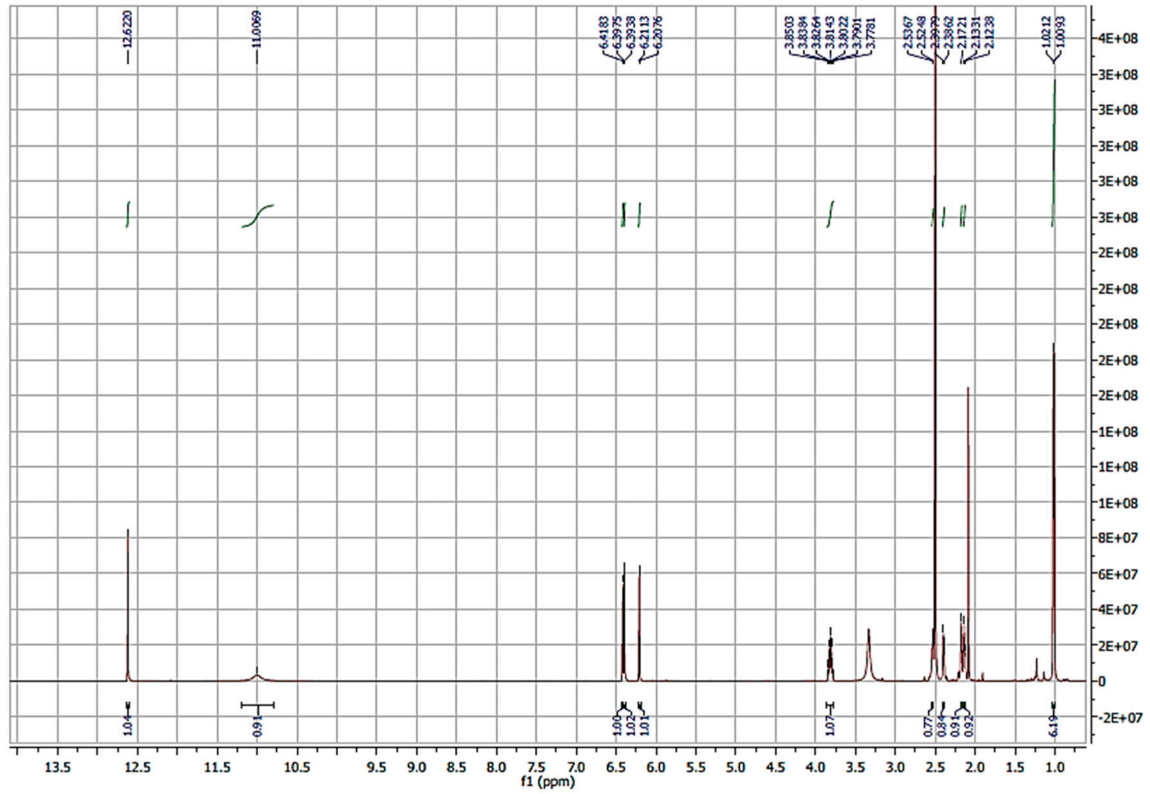

Figure S17. ^{1}H NMR (500 MHz, DMSO-d_6) spectrum of compound **19**.

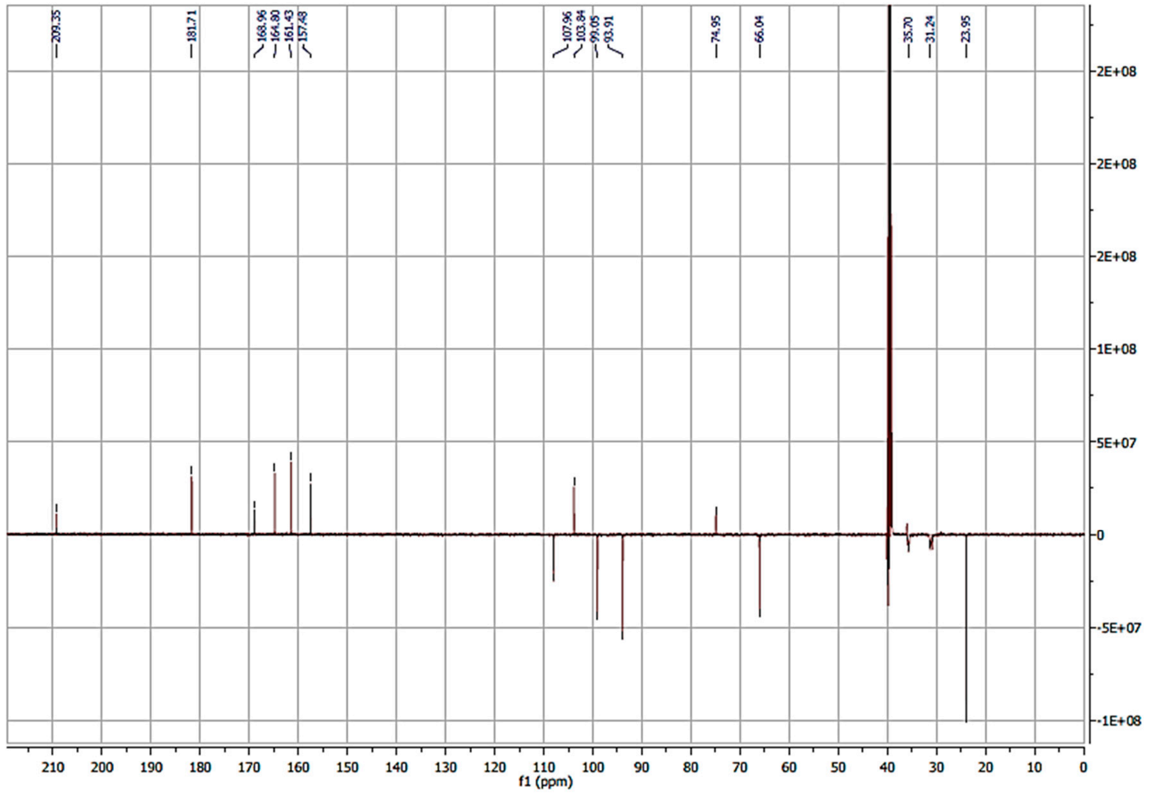

Figure S18. JMOD (125 MHz, DMSO-d_6) spectrum of compound **19**.

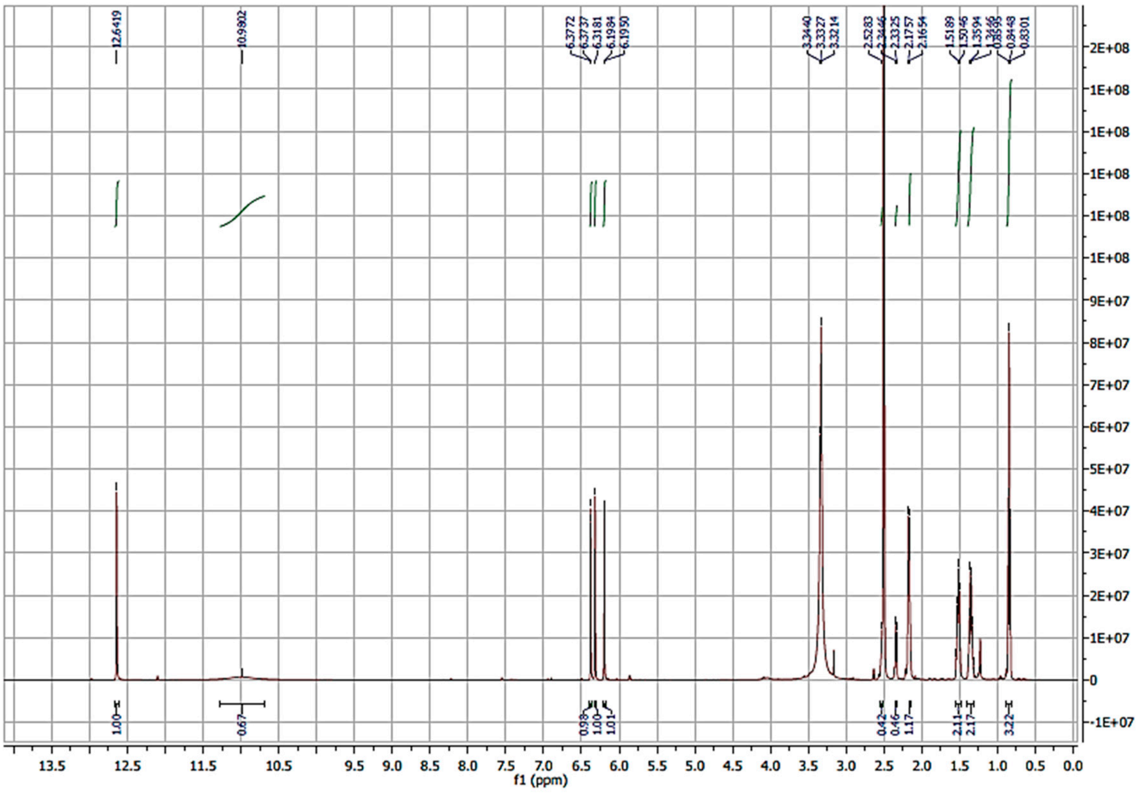

Figure S19. ^{1}H NMR (500 MHz, DMSO-d_6) spectrum of compound **20**.

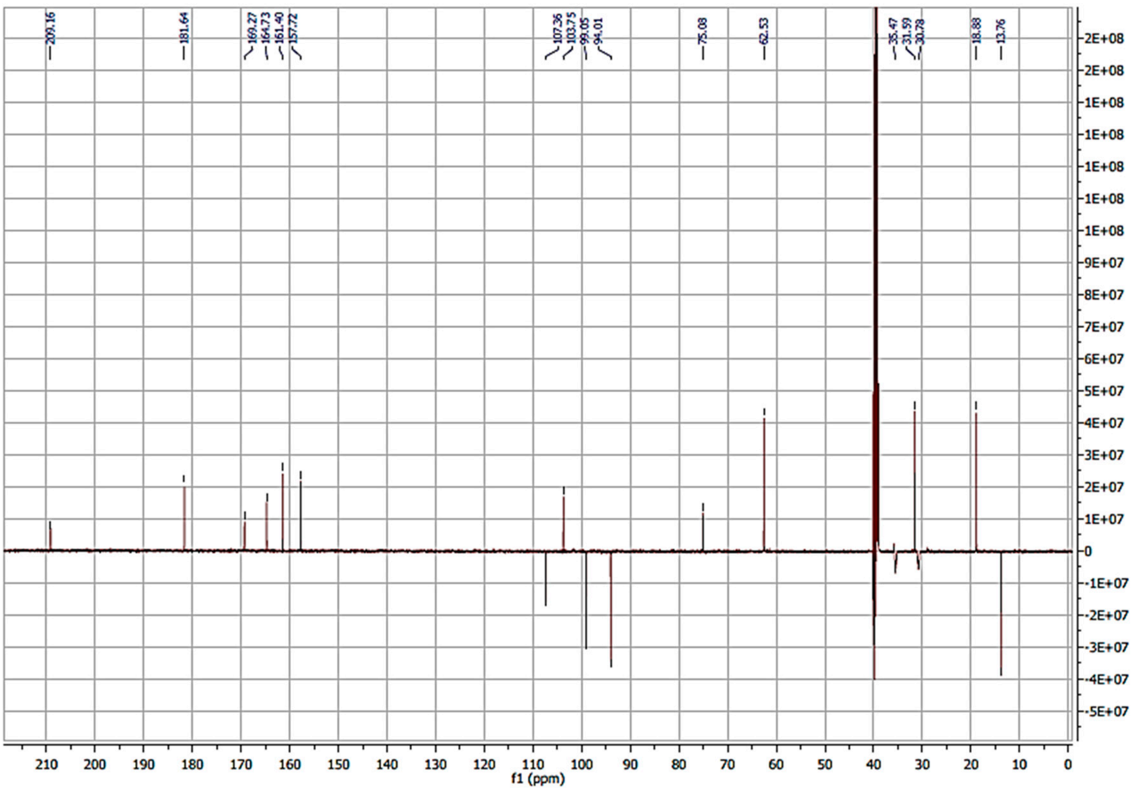

Figure S20. JMOD (125 MHz, DMSO-d_6) spectrum of compound **20**.

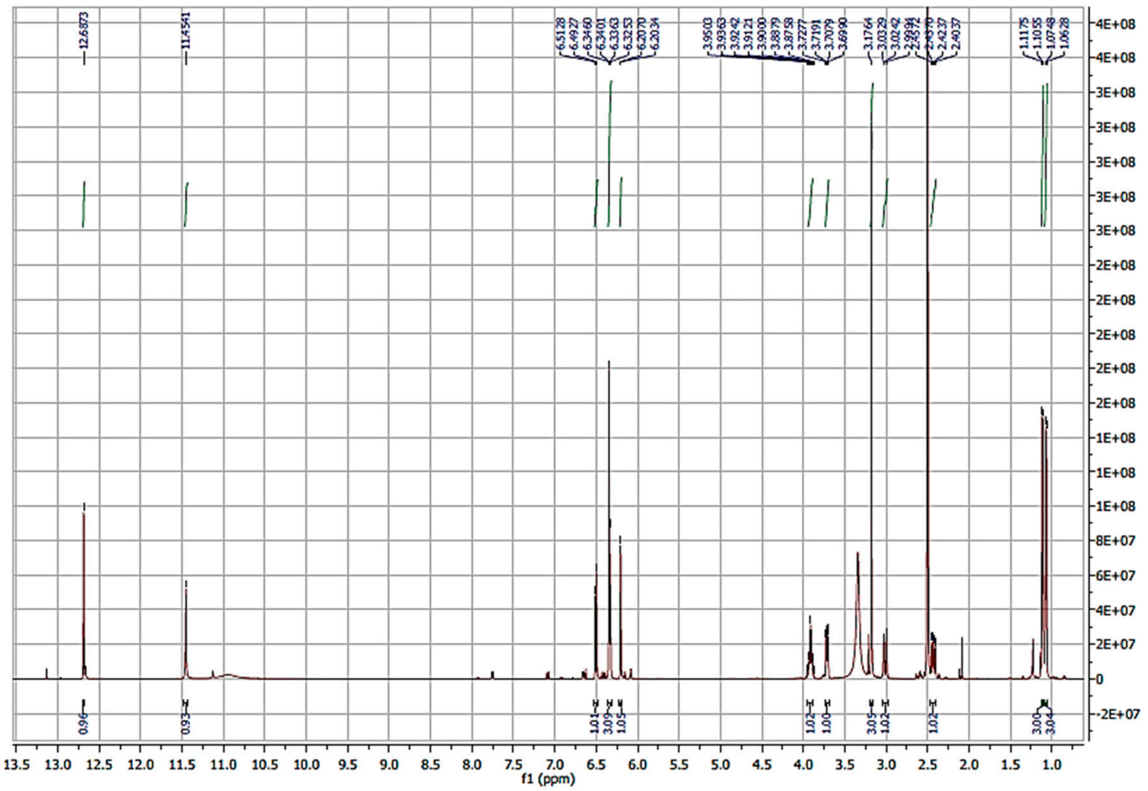

Figure S21. ^{1}H NMR (500 MHz, DMSO-d_6) spectrum of compound **21**.

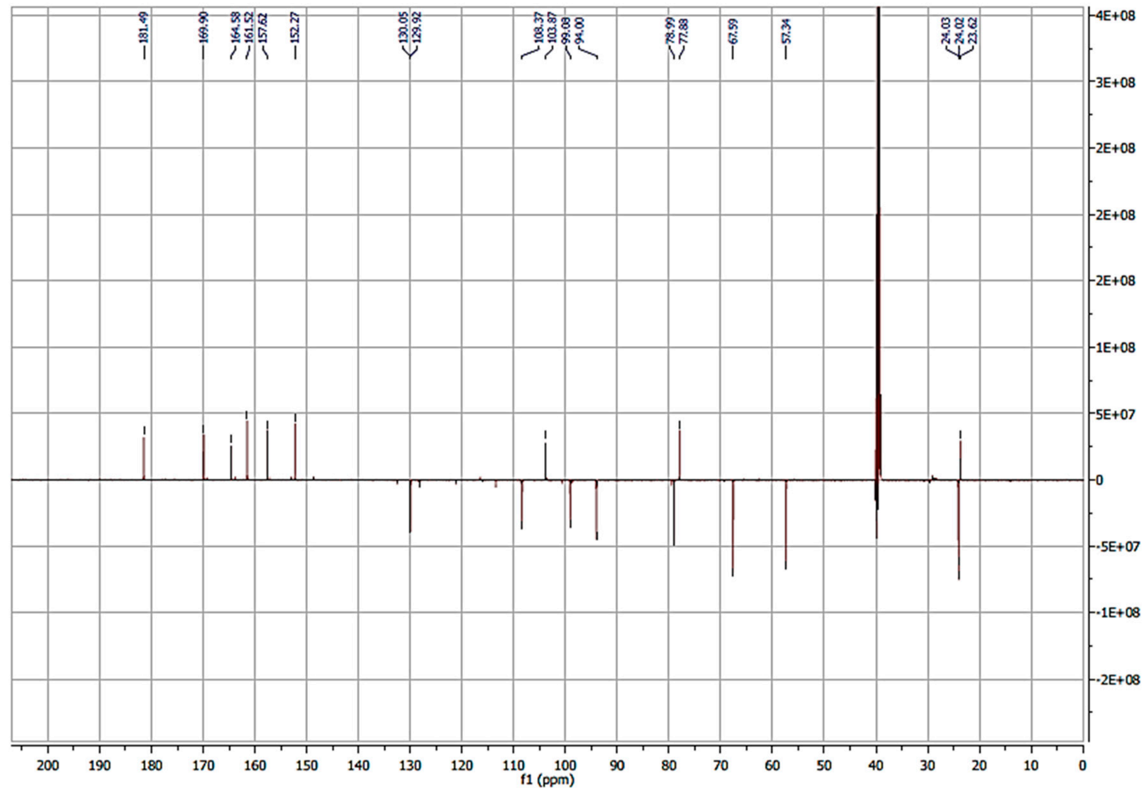

Figure S22. JMOD (125 MHz, DMSO-d_6) spectrum of compound **21**.

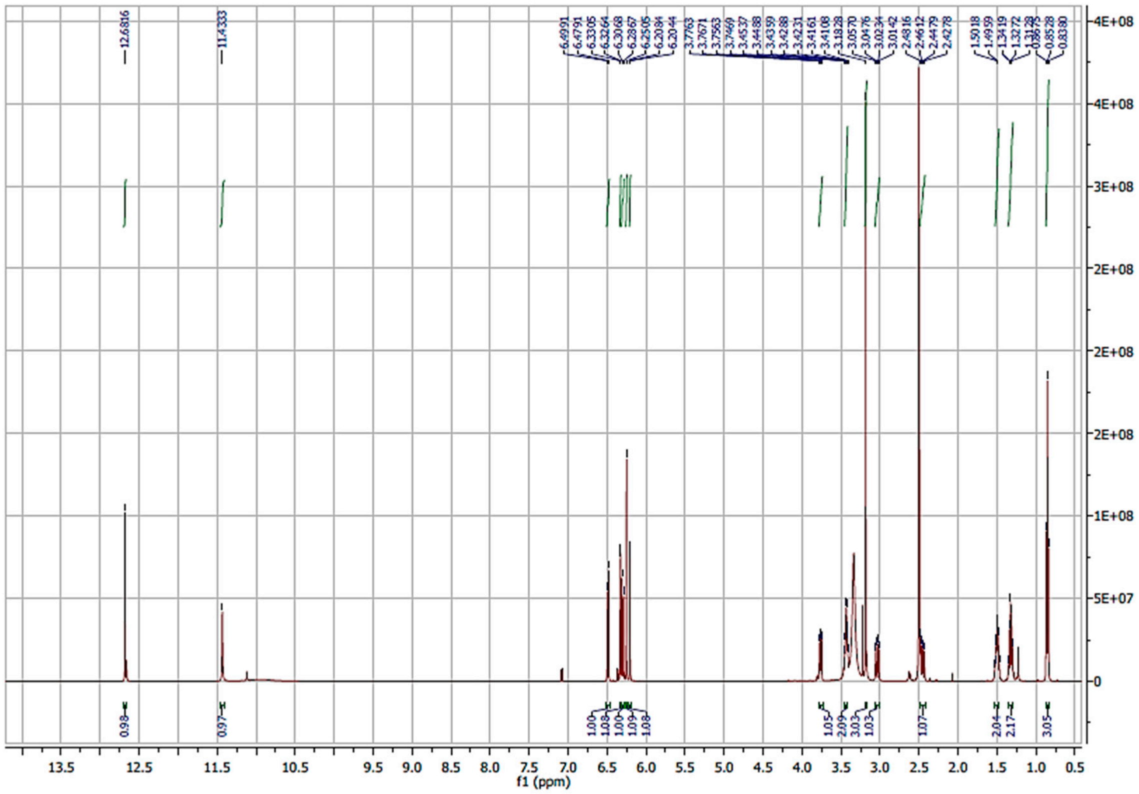

Figure S23. ^{1}H NMR (500 MHz, DMSO-d_6) spectrum of compound **22**.

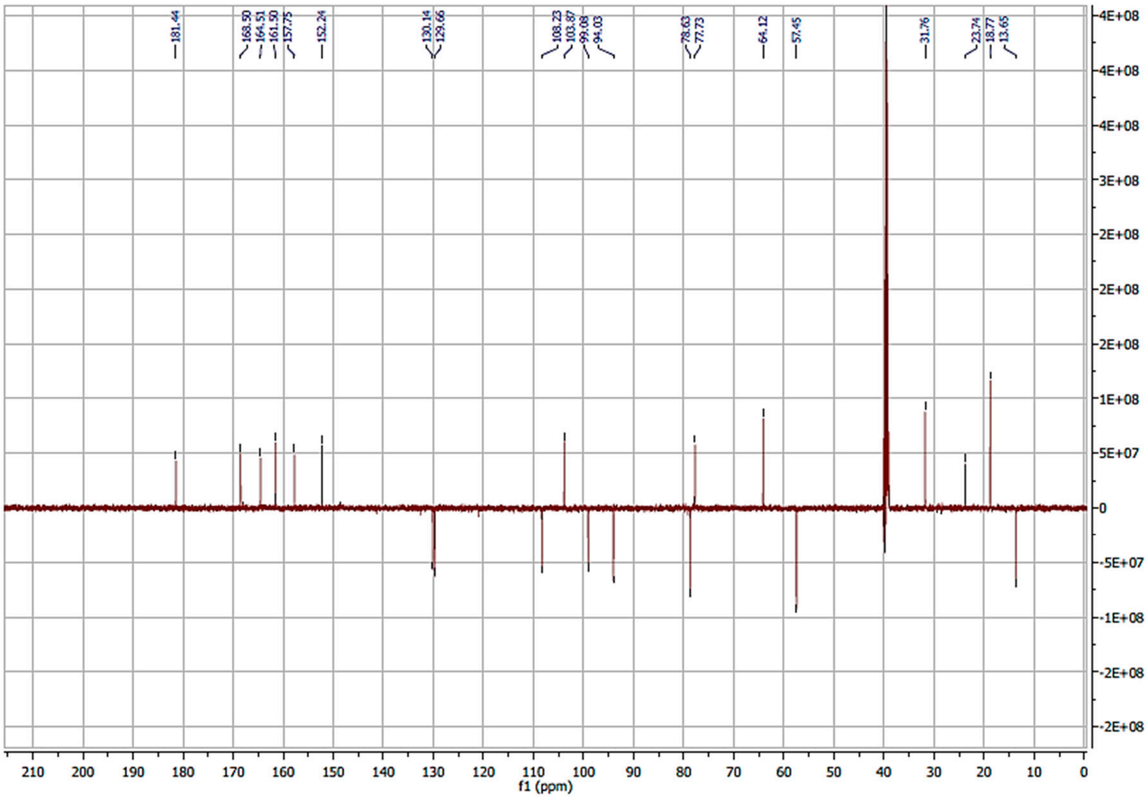

Figure S24. JMOD (125 MHz, DMSO-d_6) spectrum of compound **22**.

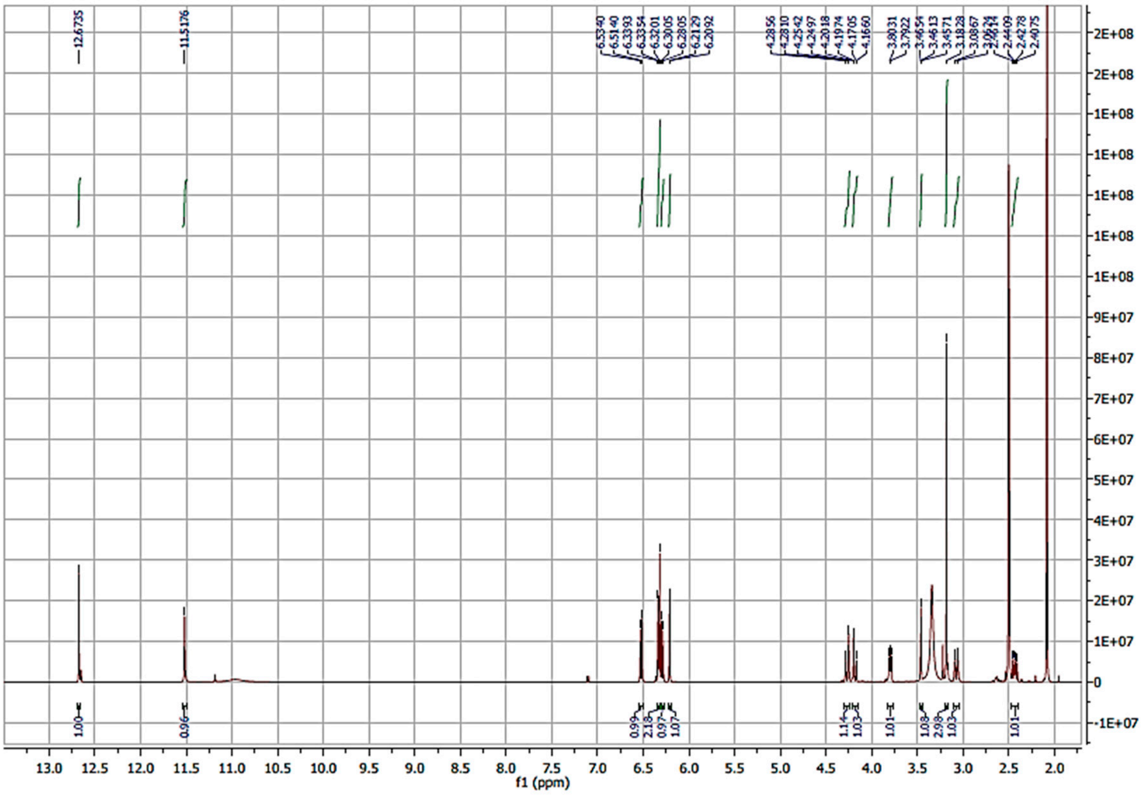

Figure S25. ^{1}H NMR (500 MHz, DMSO-d_6) spectrum of compound **23**.

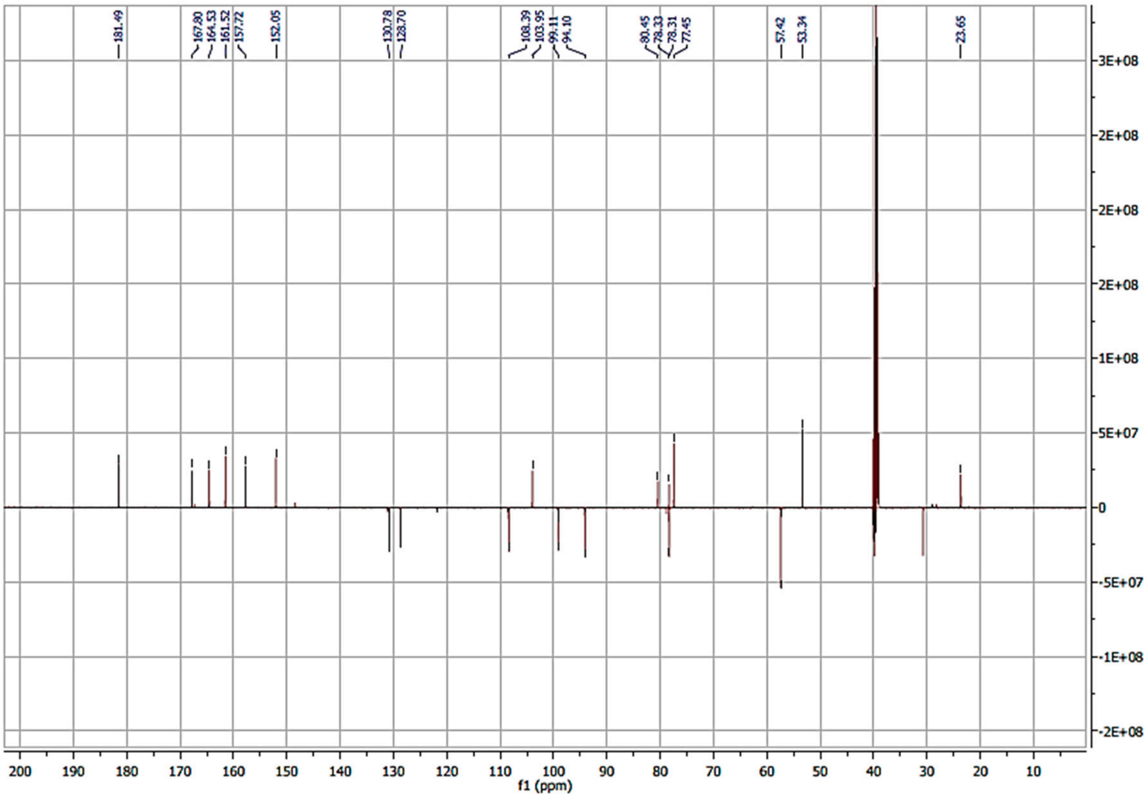

Figure S26. JMOD (125 MHz, DMSO-d_6) spectrum of compound **23**.

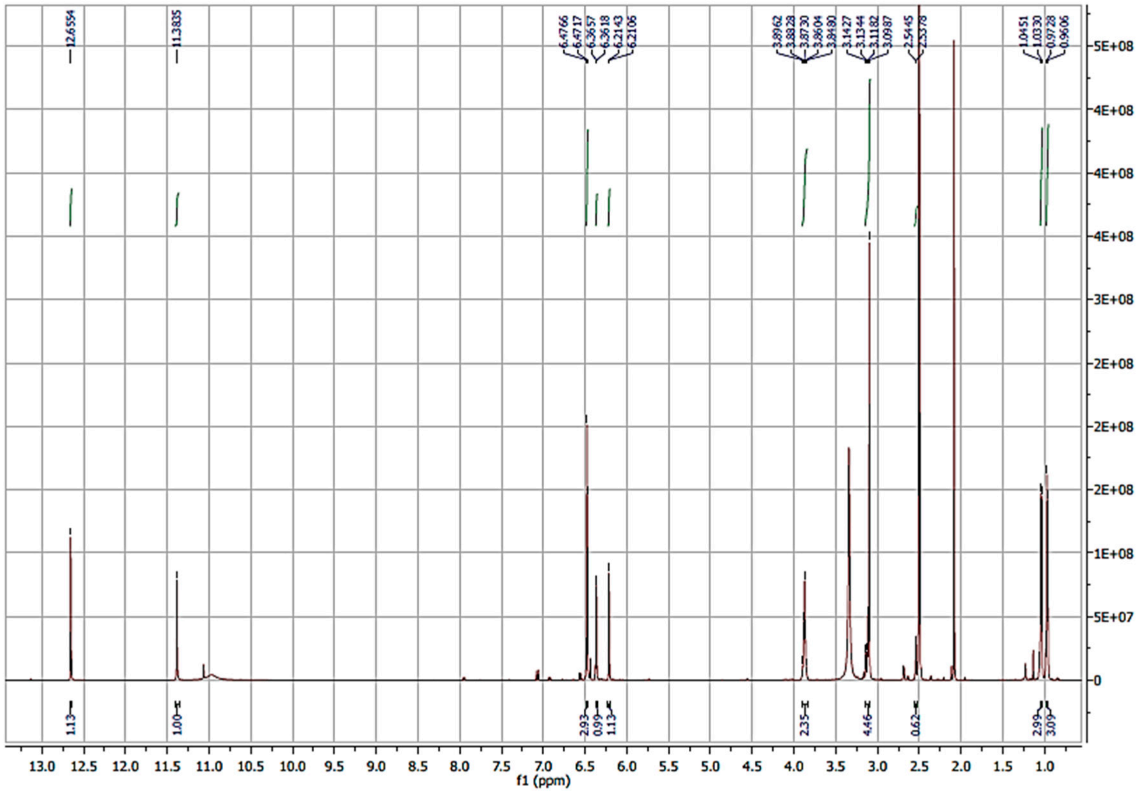

Figure S27. ^{1}H NMR (500 MHz, DMSO-d_6) spectrum of compound **24**.

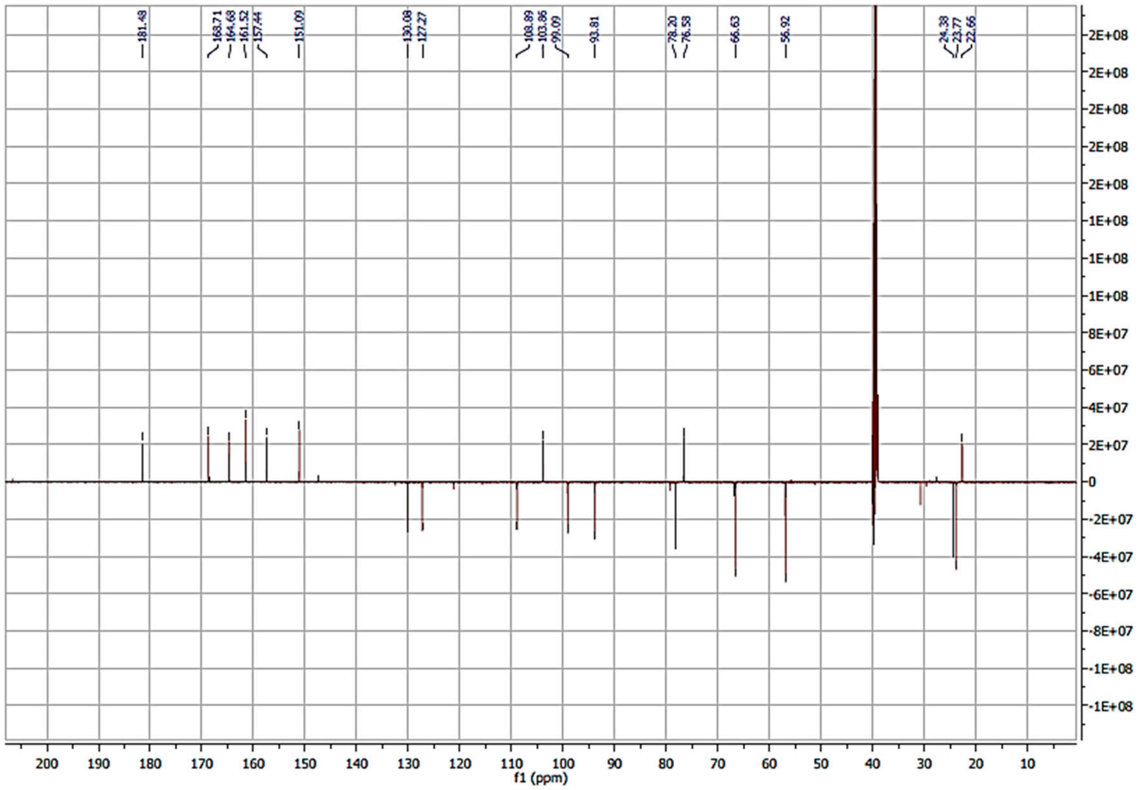

Figure S28. JMOD (125 MHz, DMSO-d_6) spectrum of compound **24**.

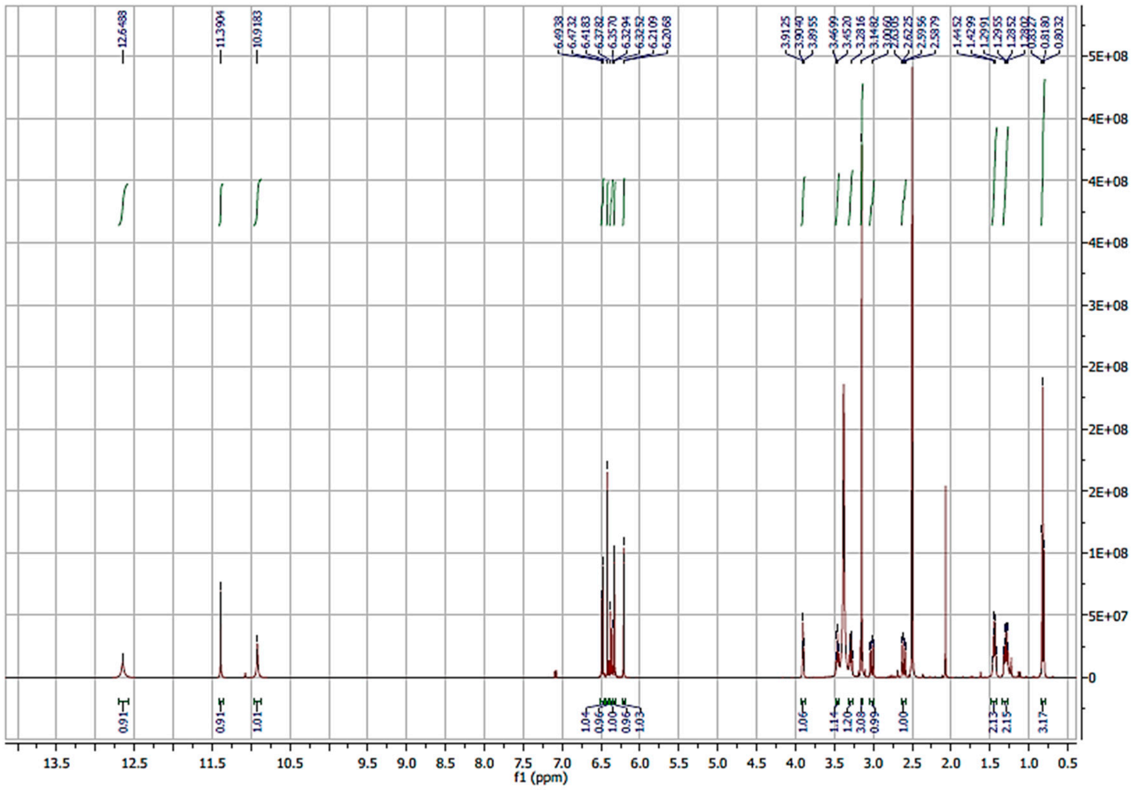

Figure S29. ^{1}H NMR (500 MHz, DMSO-d_6) spectrum of compound **25**.

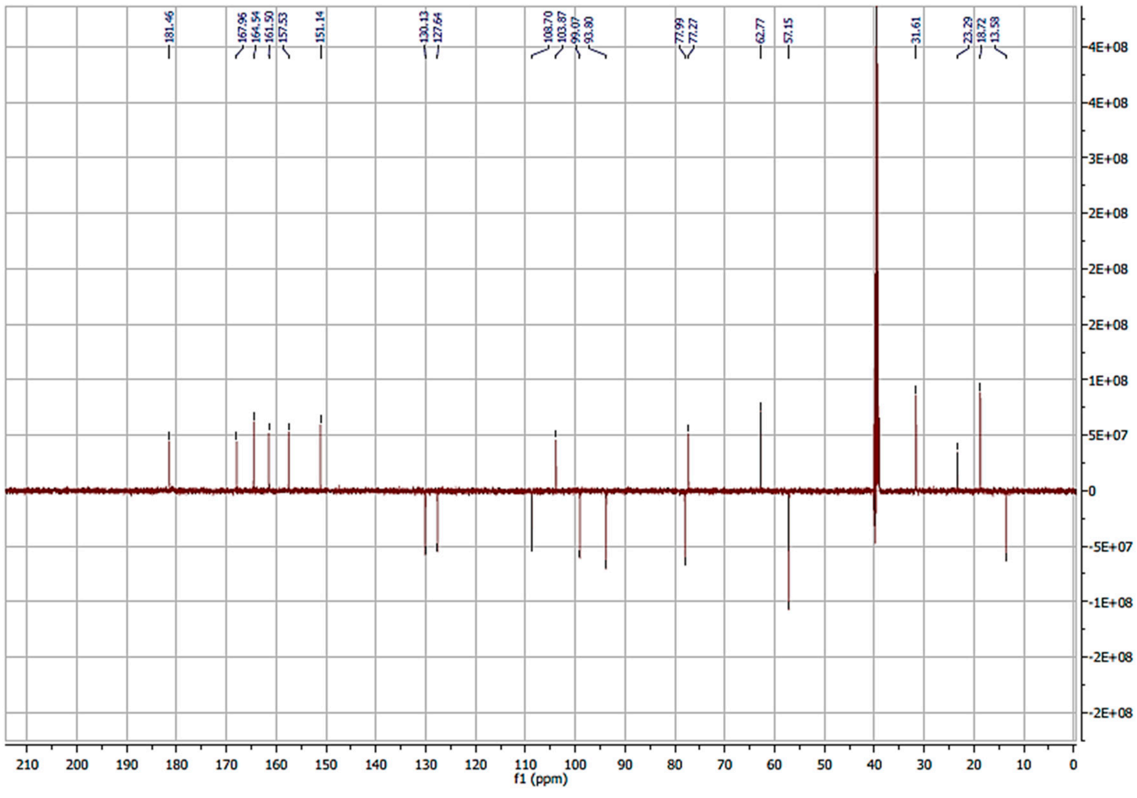

Figure S30. JMOD (125 MHz, DMSO-d_6) spectrum of compound **25**.

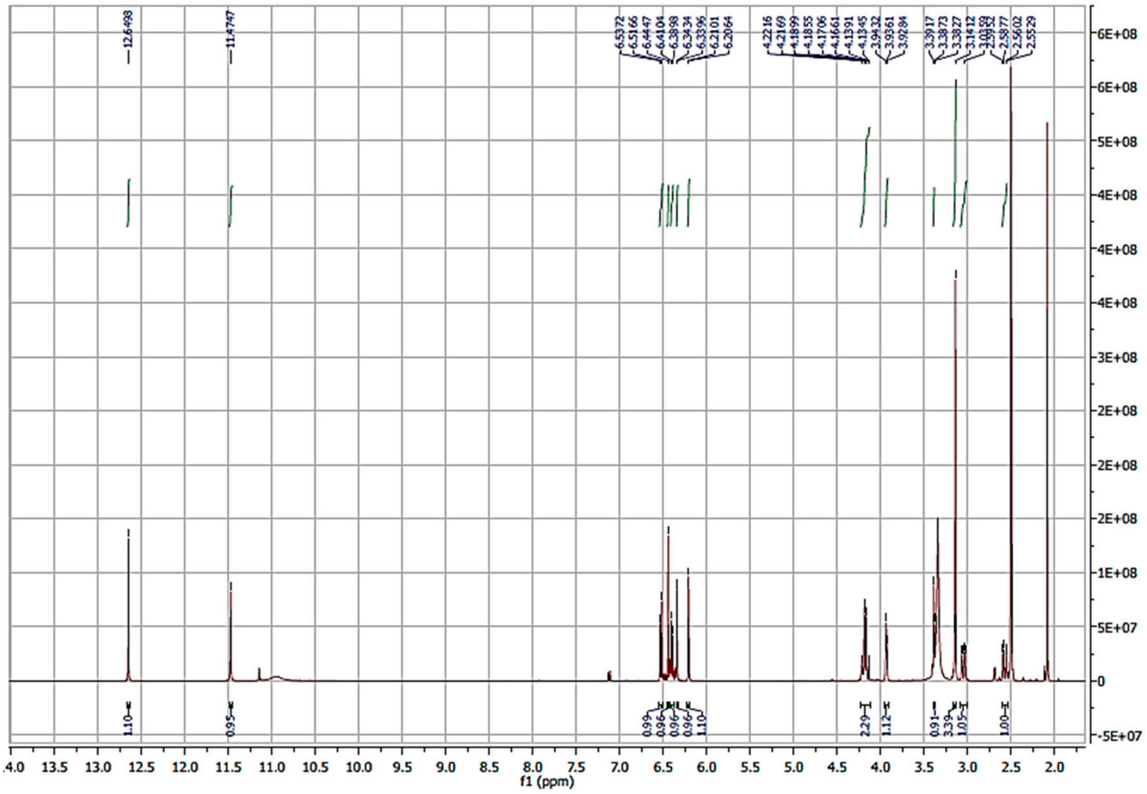

Figure S31. ^{1}H NMR (500 MHz, DMSO-d_6) spectrum of compound **26**.

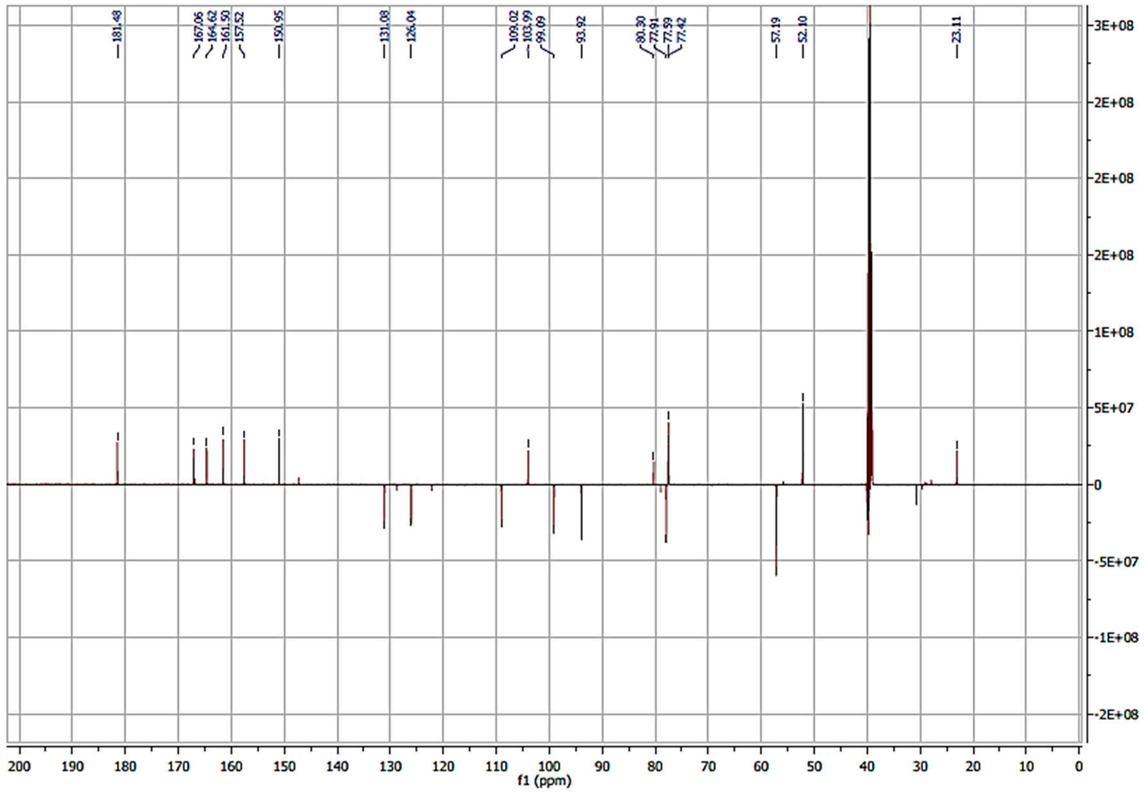

Figure S32. JMOD (125 MHz, DMSO-d_6) spectrum of compound **26**.

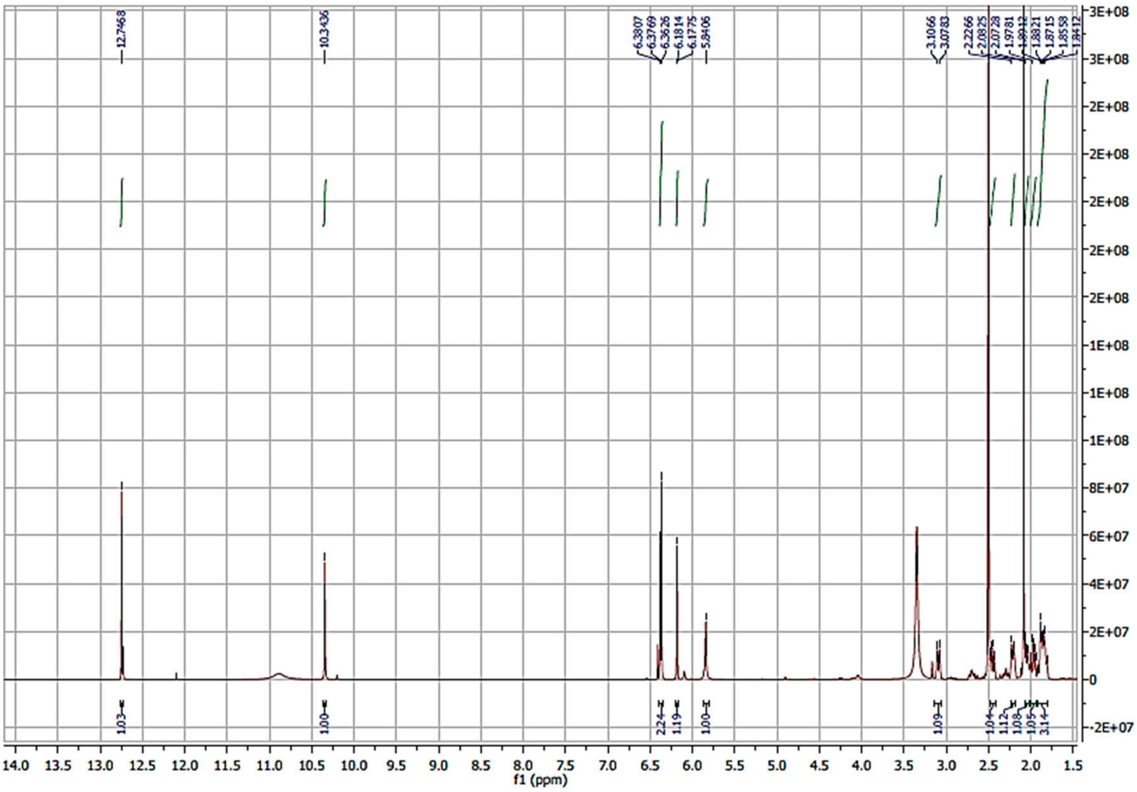

Figure S33. ^{1}H NMR (500 MHz, DMSO-d_6) spectrum of compound **27**.

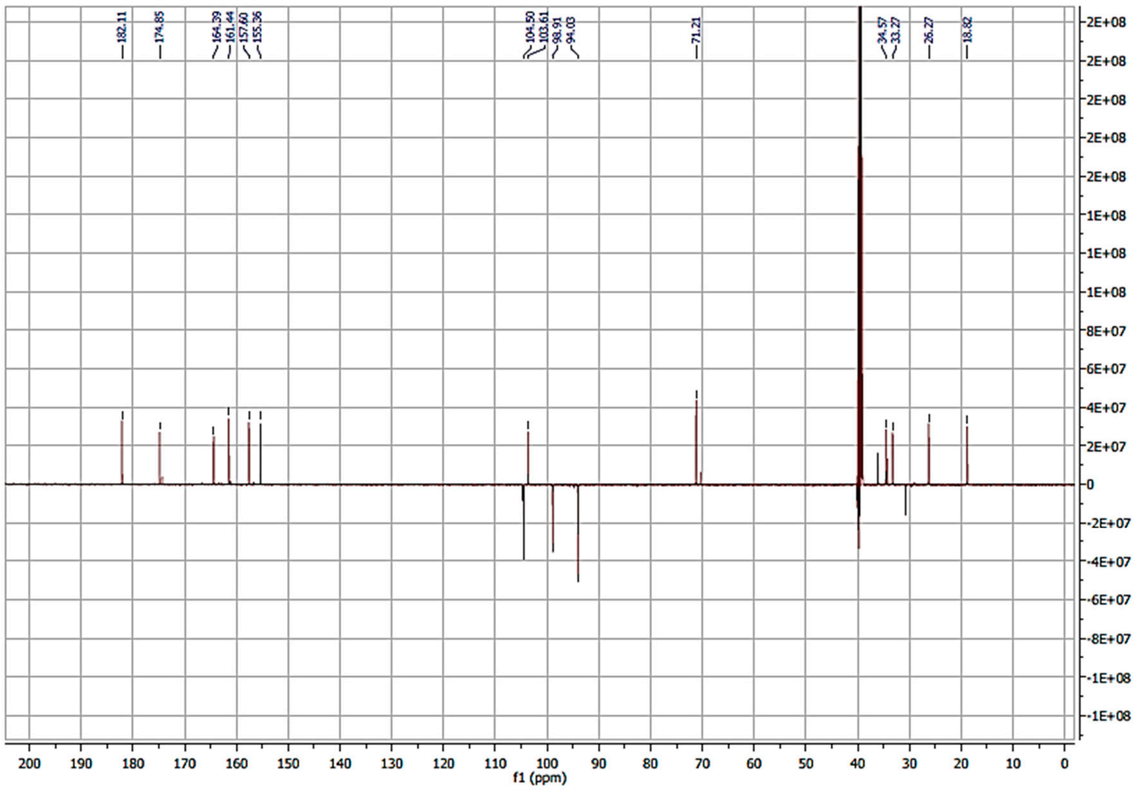

Figure S34. JMOD (125 MHz, DMSO-d_6) spectrum of compound **27**.

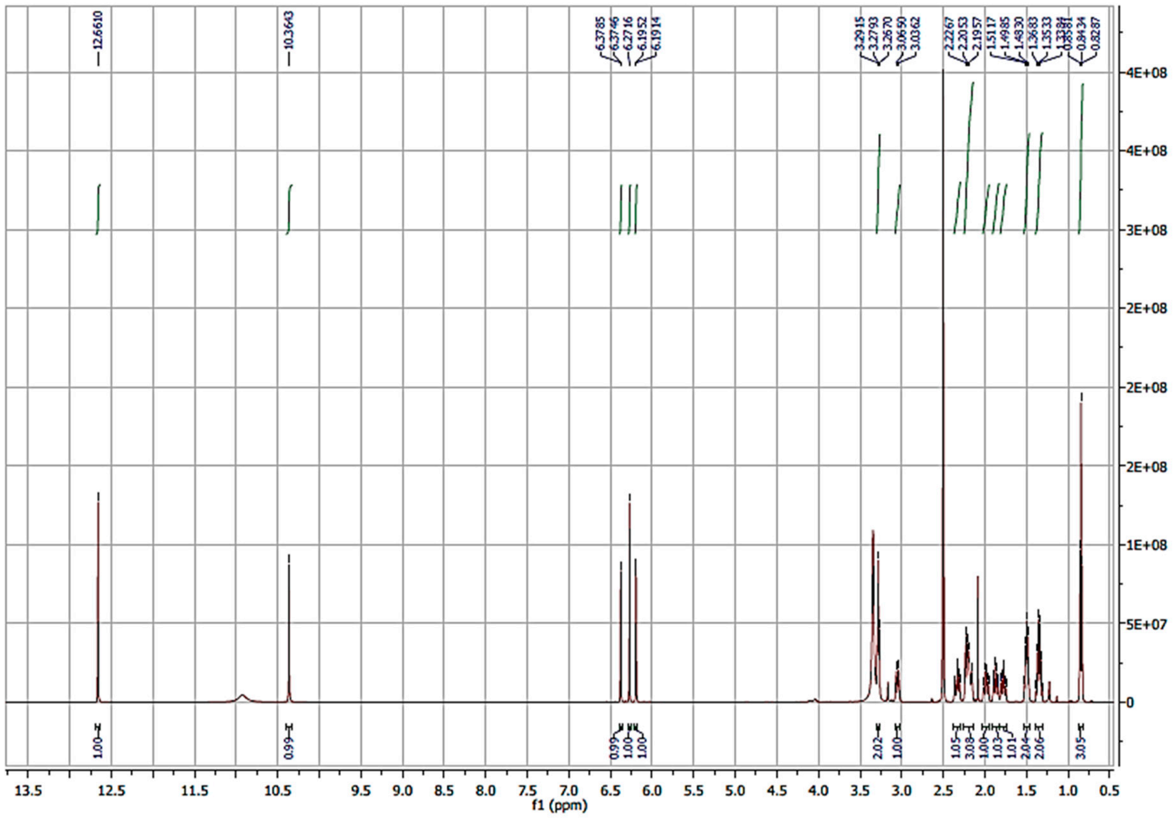

Figure S35. ^{1}H NMR (500 MHz, DMSO-d_6) spectrum of compound **28**.

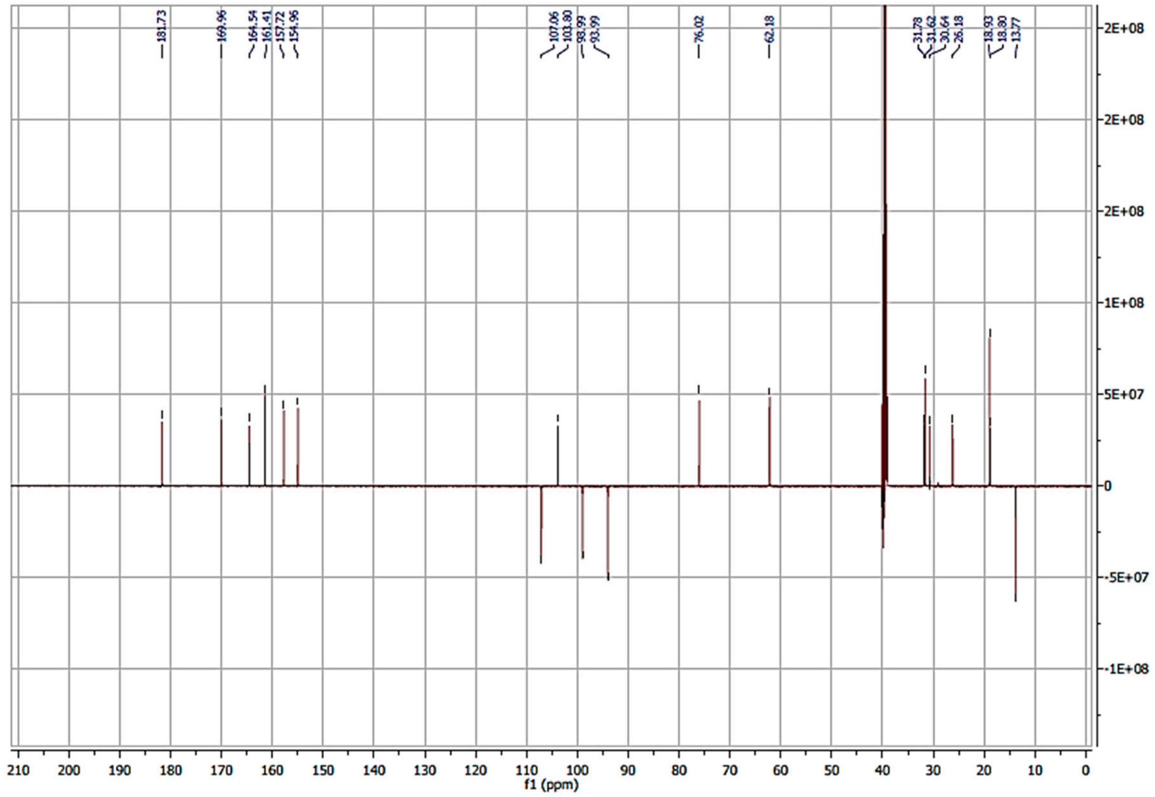

Figure S36. JMOD (125 MHz, DMSO-d_6) spectrum of compound **28**.

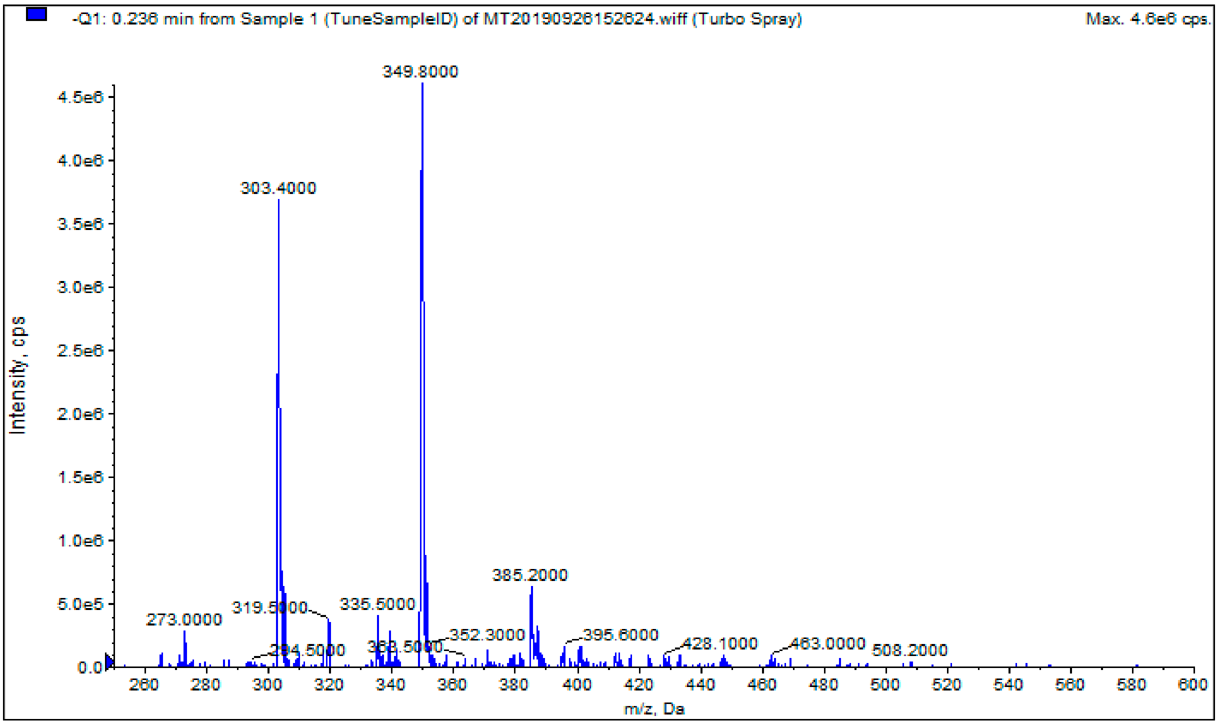

Figure S37. Mass spectrum of compound **10** recorded in negative ionization mode. (solvent: CH_3CN + 1% HCOOH)

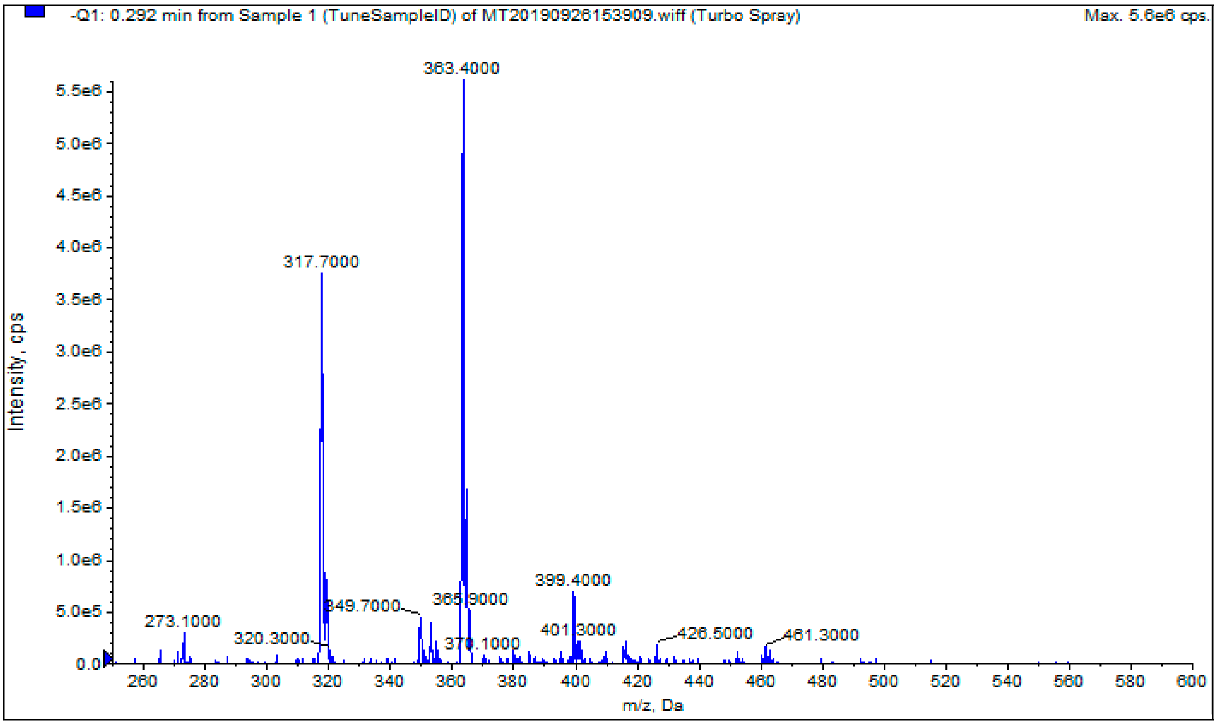

Figure S38. Mass spectrum of compound **11** recorded in negative ionization mode. (solvent: CH_3CN + 1% HCOOH)

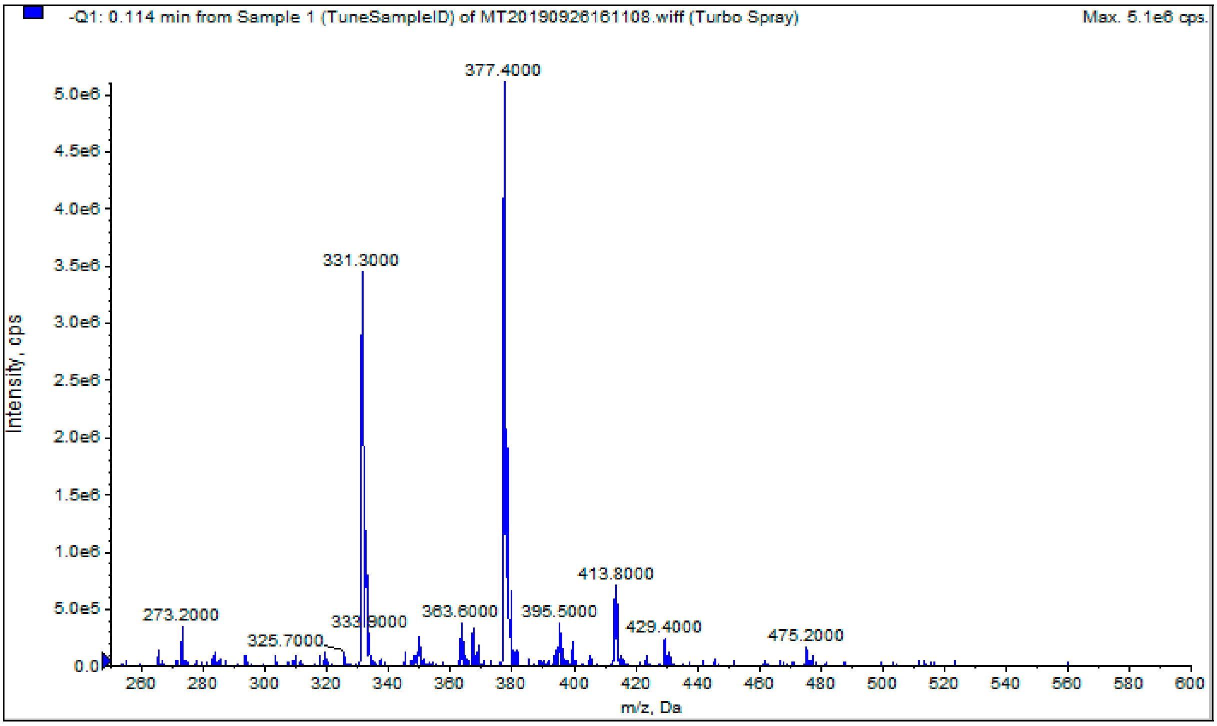

Figure S39. Mass spectrum of compound **12** recorded in negative ionization mode. (solvent: CH_3CN + 1% HCOOH)

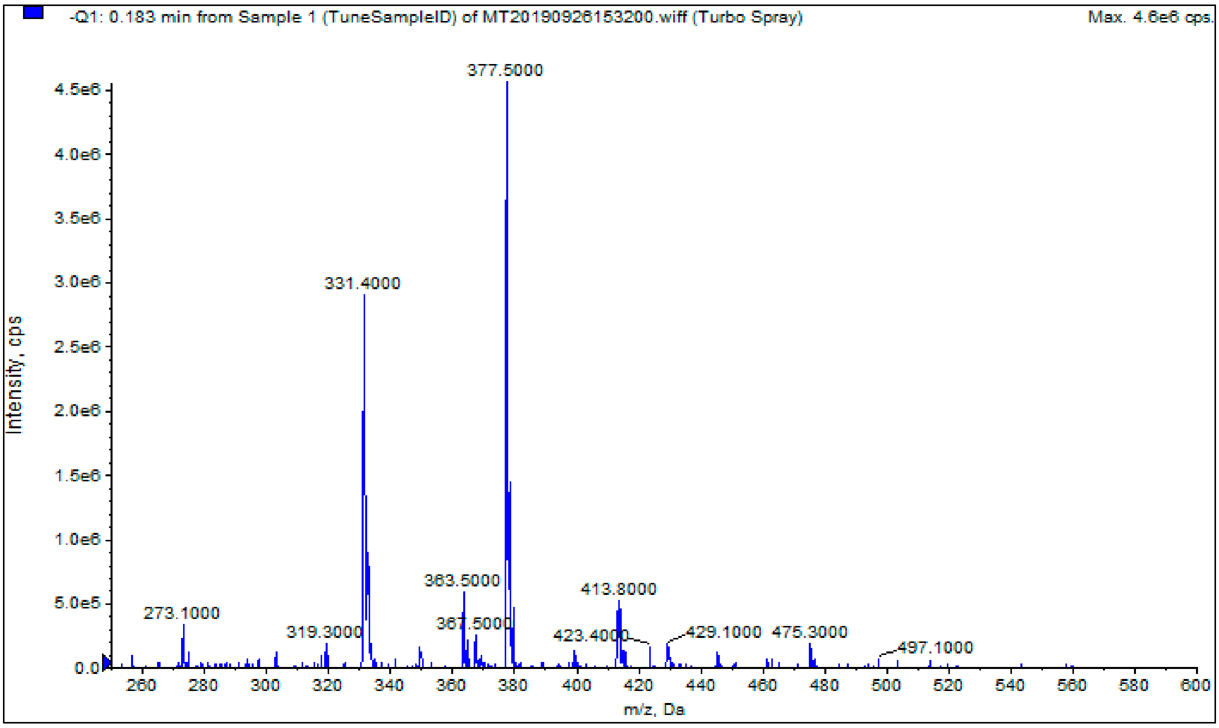

Figure S40. Mass spectrum of compound **13** recorded in negative ionization mode. (solvent: CH_3CN + 1% HCOOH)

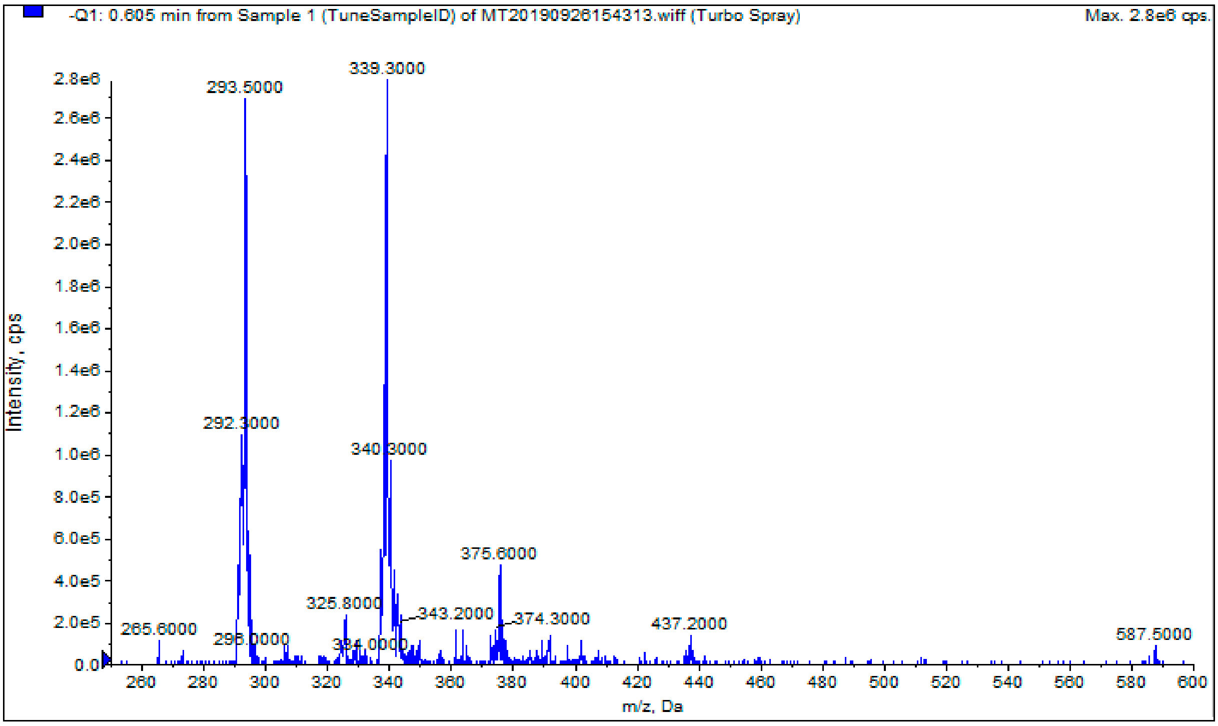

Figure S41. Mass spectrum of compound **15** recorded in negative ionization mode. (solvent: CH_3CN + 1% HCOOH)

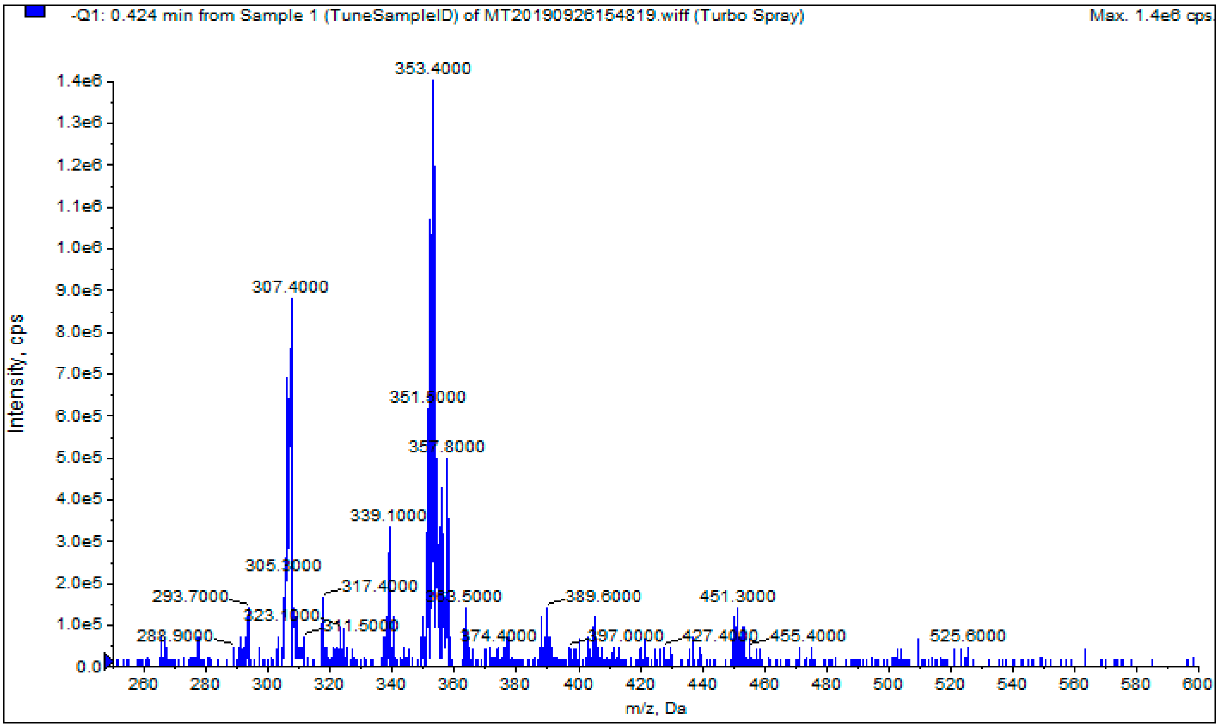

Figure S42. Mass spectrum of compound **16** recorded in negative ionization mode. (solvent: CH_3CN + 1% HCOOH)

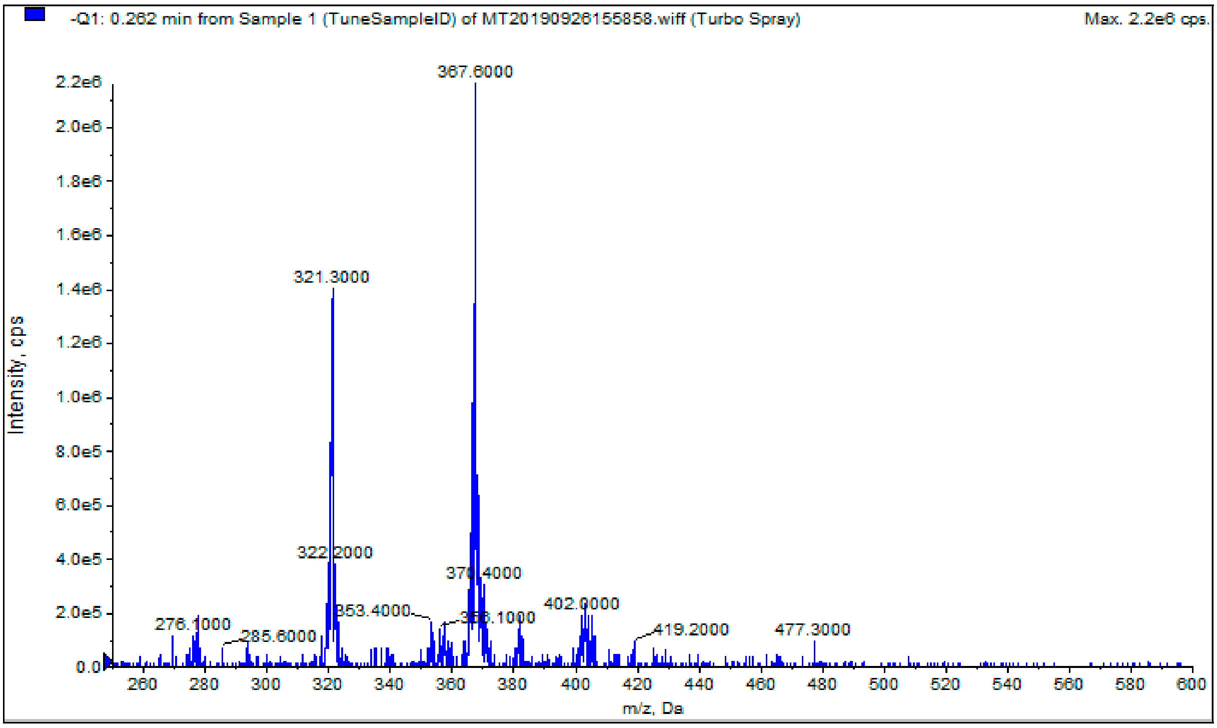

Figure S43. Mass spectrum of compound **17** recorded in negative ionization mode. (solvent: CH_3CN + 1% HCOOH)

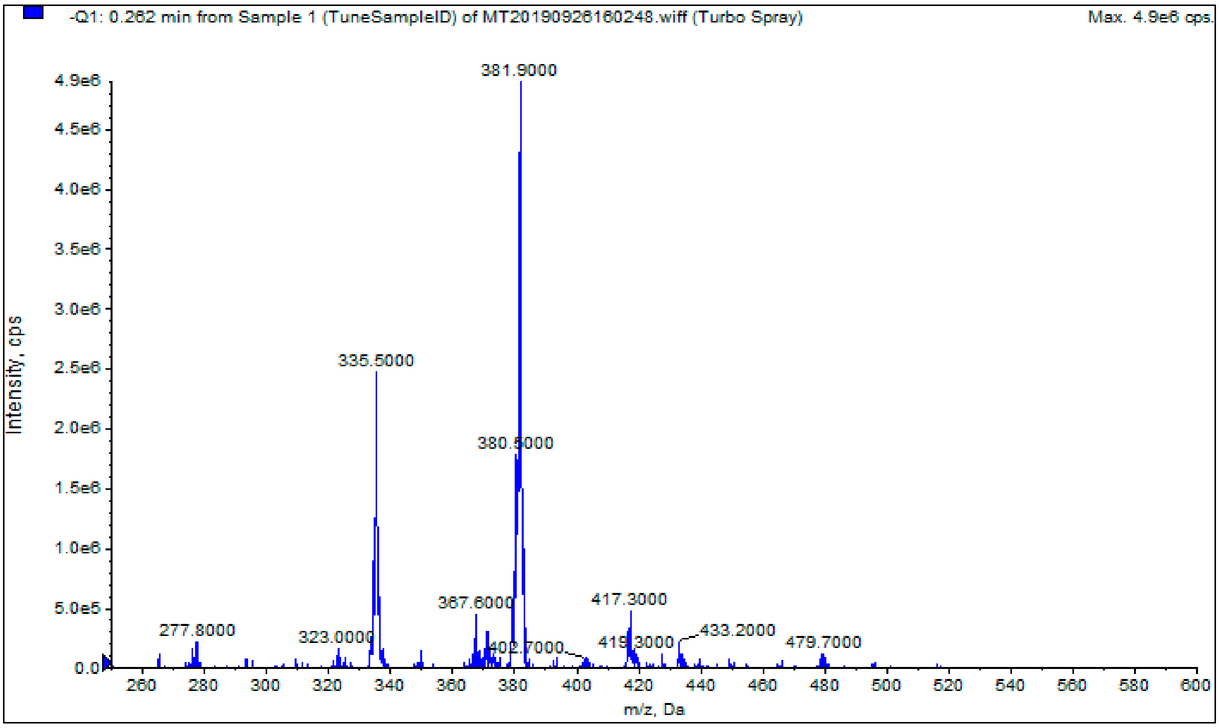

Figure S44. Mass spectrum of compound **18** recorded in negative ionization mode. (solvent: CH_3CN + 1% HCOOH)

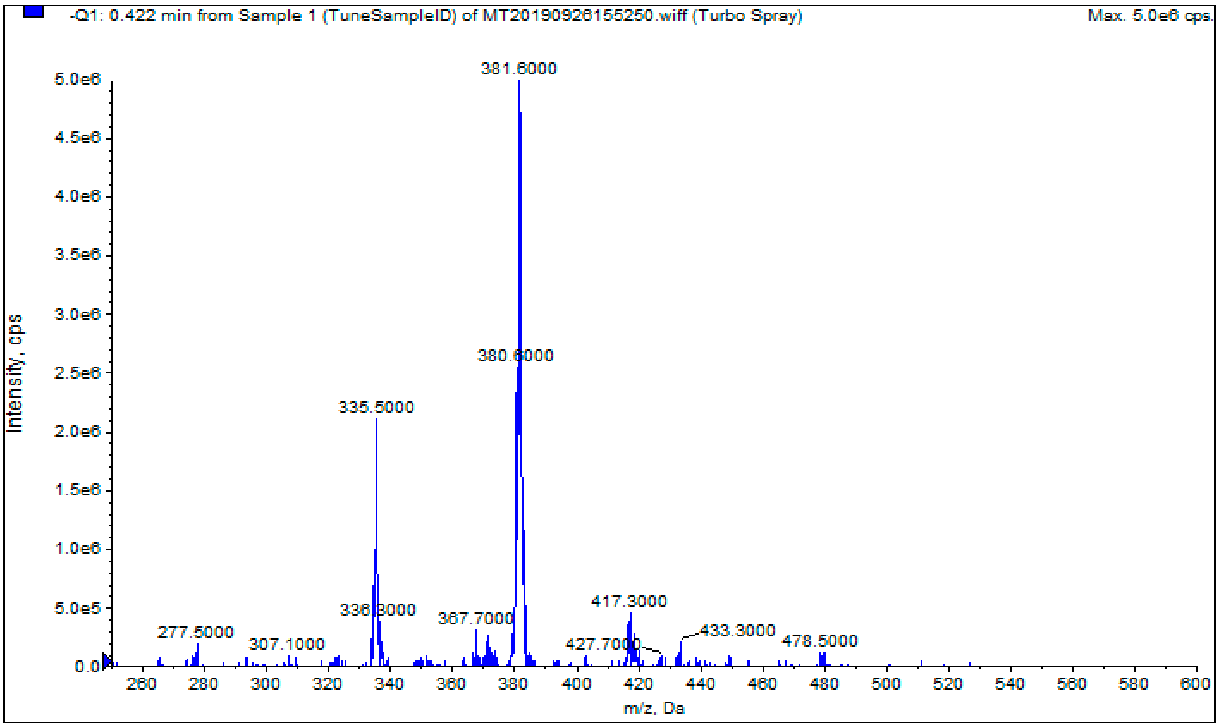

Figure S45. Mass spectrum of compound **19** recorded in negative ionization mode. (solvent: CH_3CN + 1% HCOOH)

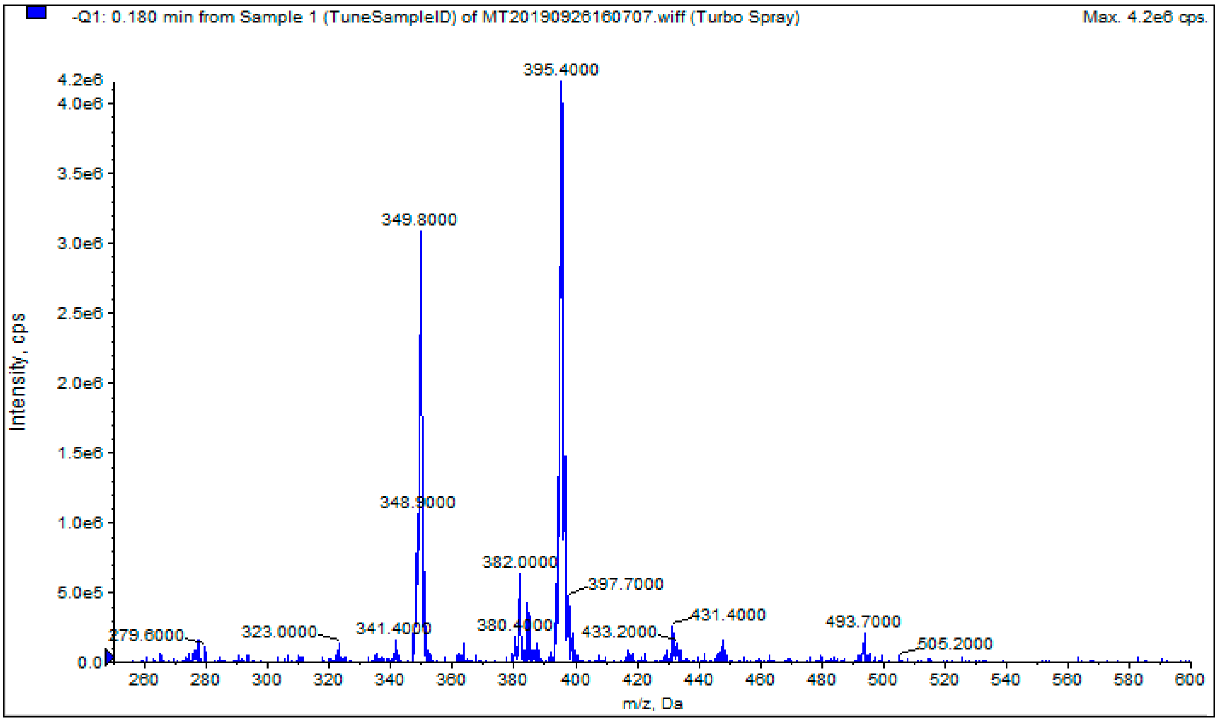

Figure S46. Mass spectrum of compound **20** recorded in negative ionization mode. (solvent: CH_3CN + 1% HCOOH)

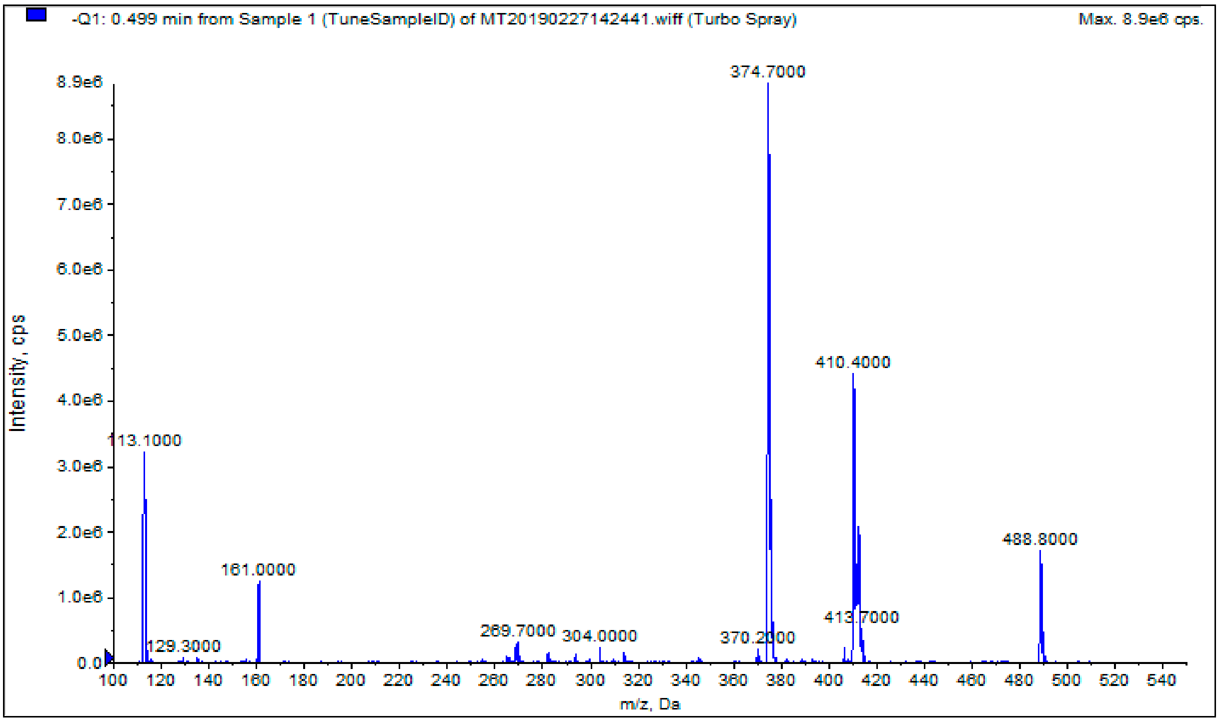

Figure S47. Mass spectrum of racemate **21** recorded in negative ionization mode. (solvent: CH_3CN)

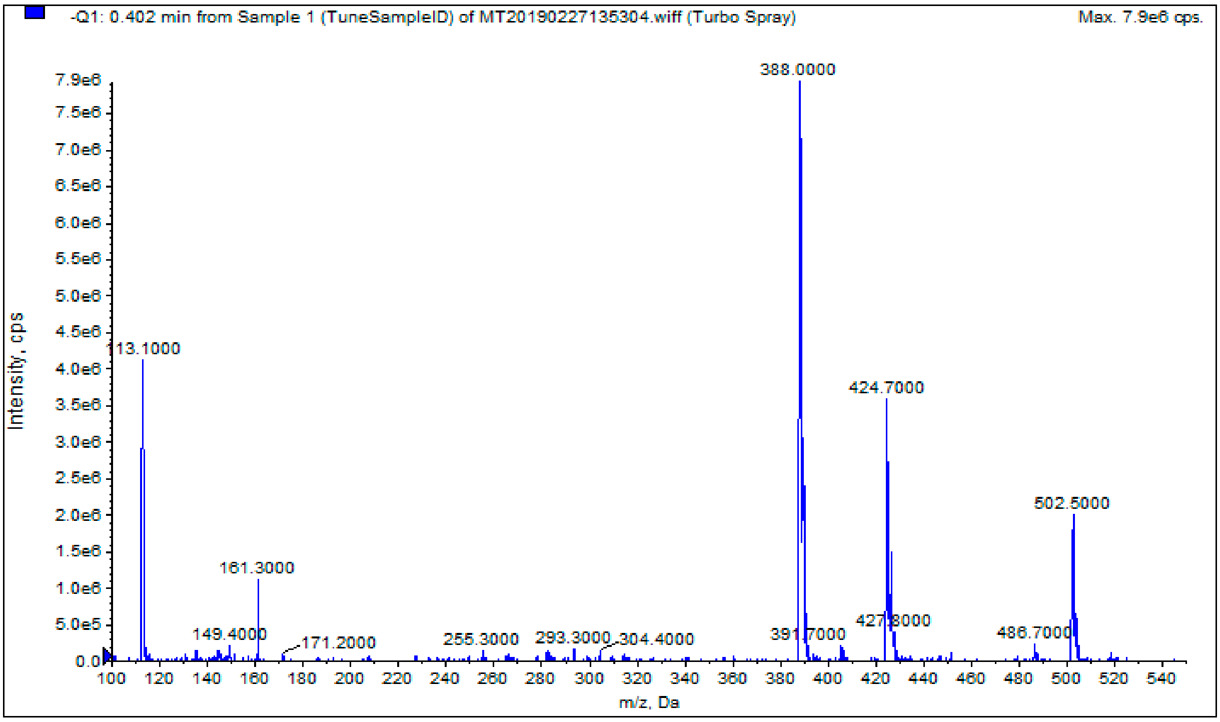

Figure S48. Mass spectrum of racemate **22** recorded in negative ionization mode. (solvent: CH_3CN)

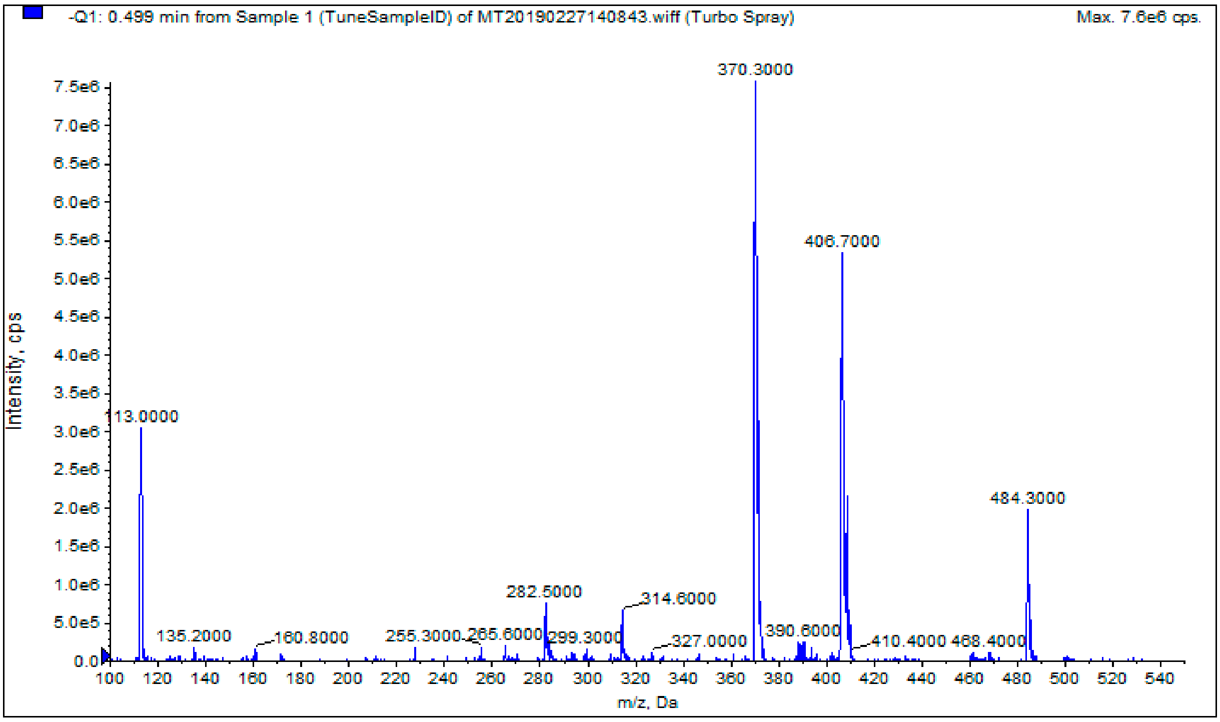

Figure S49. Mass spectrum of racemate **23** recorded in negative ionization mode. (solvent: CH_3CN)

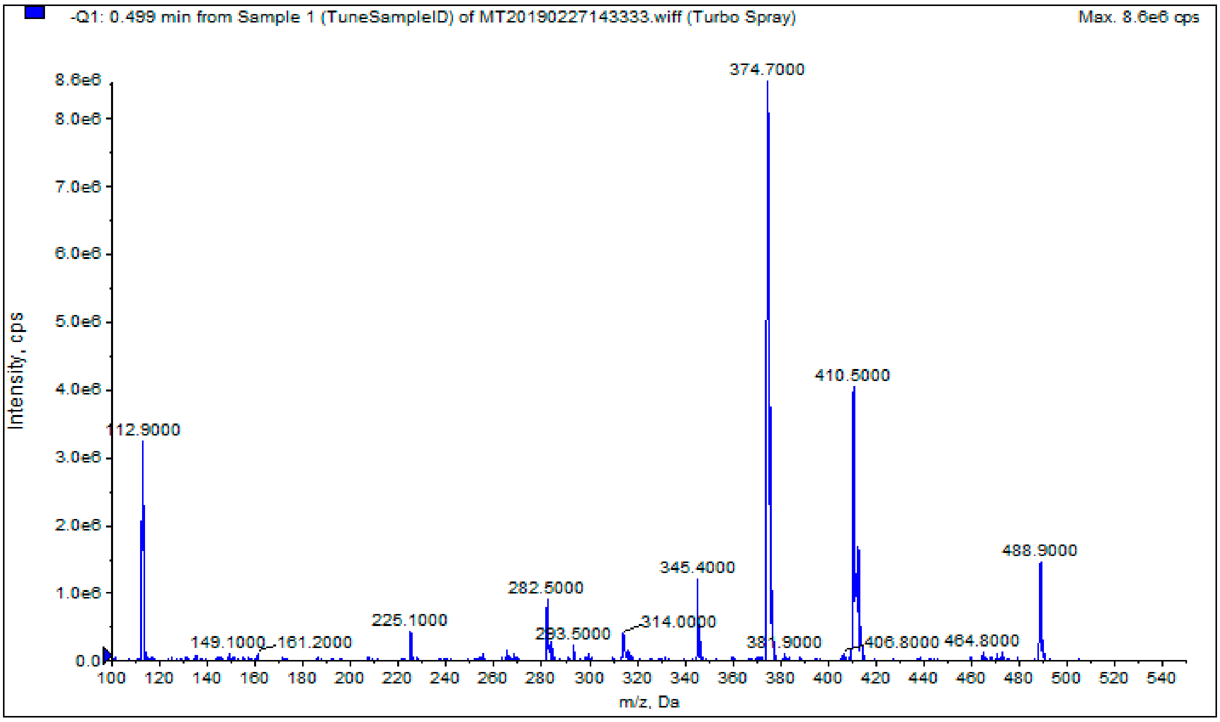

Figure S50. Mass spectrum of racemate **24** recorded in negative ionization mode. (solvent: CH_3CN)

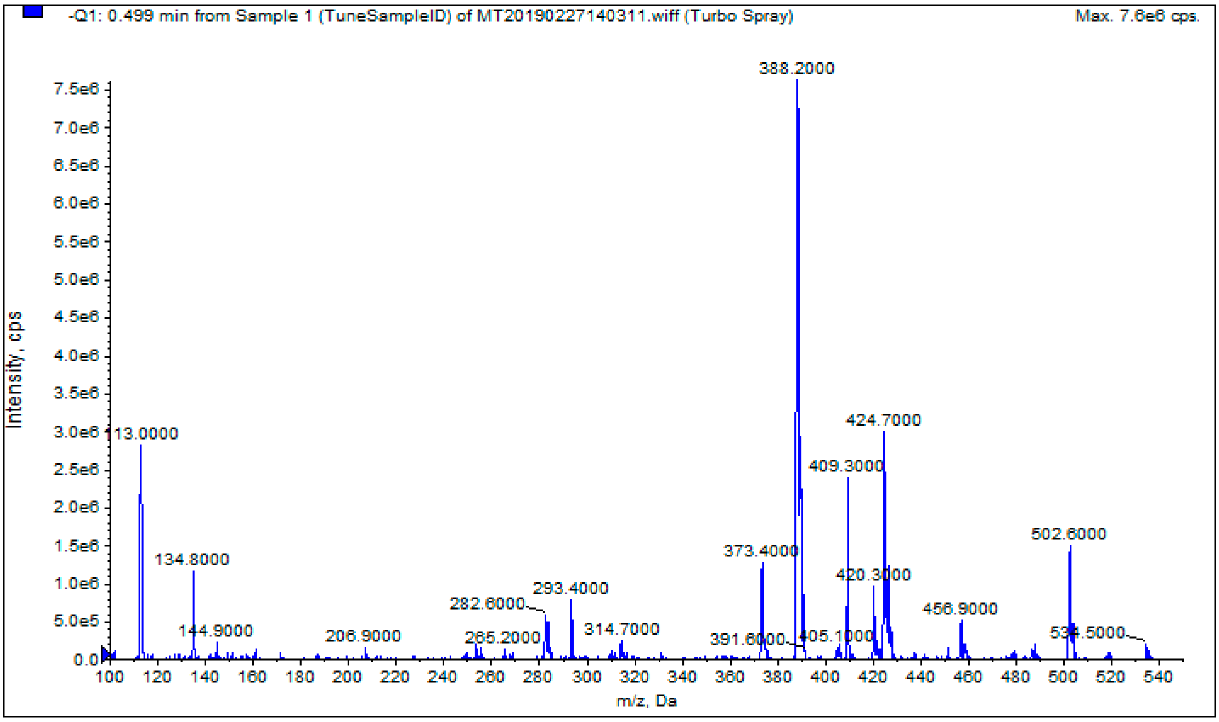

Figure S51. Mass spectrum of racemate **25** recorded in negative ionization mode. (solvent: CH_3CN)

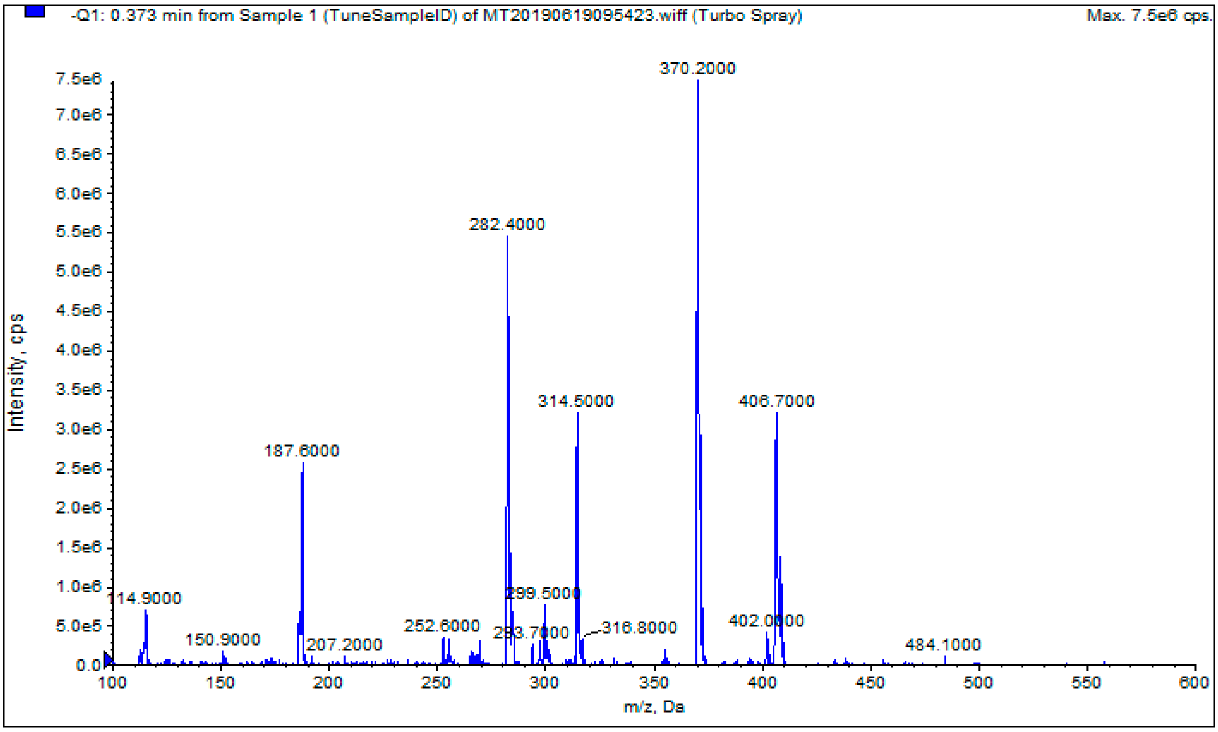

Figure S52. Mass spectrum of racemate **26** recorded in negative ionization mode. (solvent: CH_3CN)

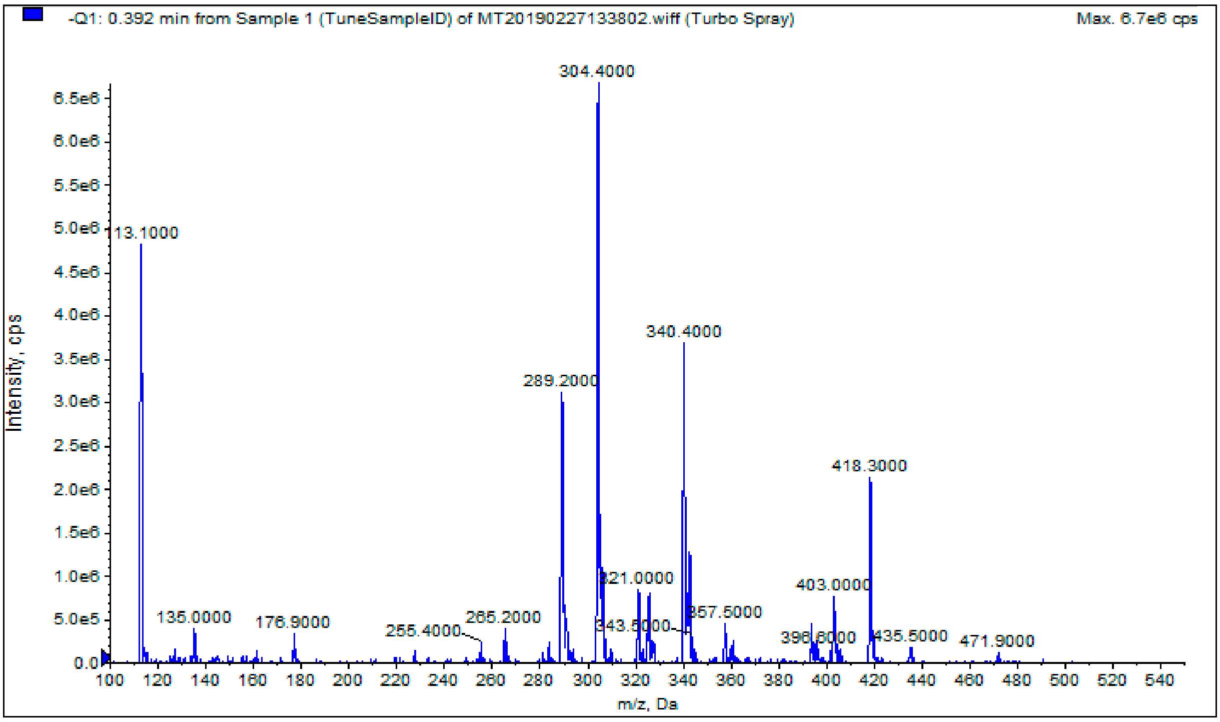

Figure S53. Mass spectrum of racemate **27** recorded in negative ionization mode. (solvent: CH_3CN)

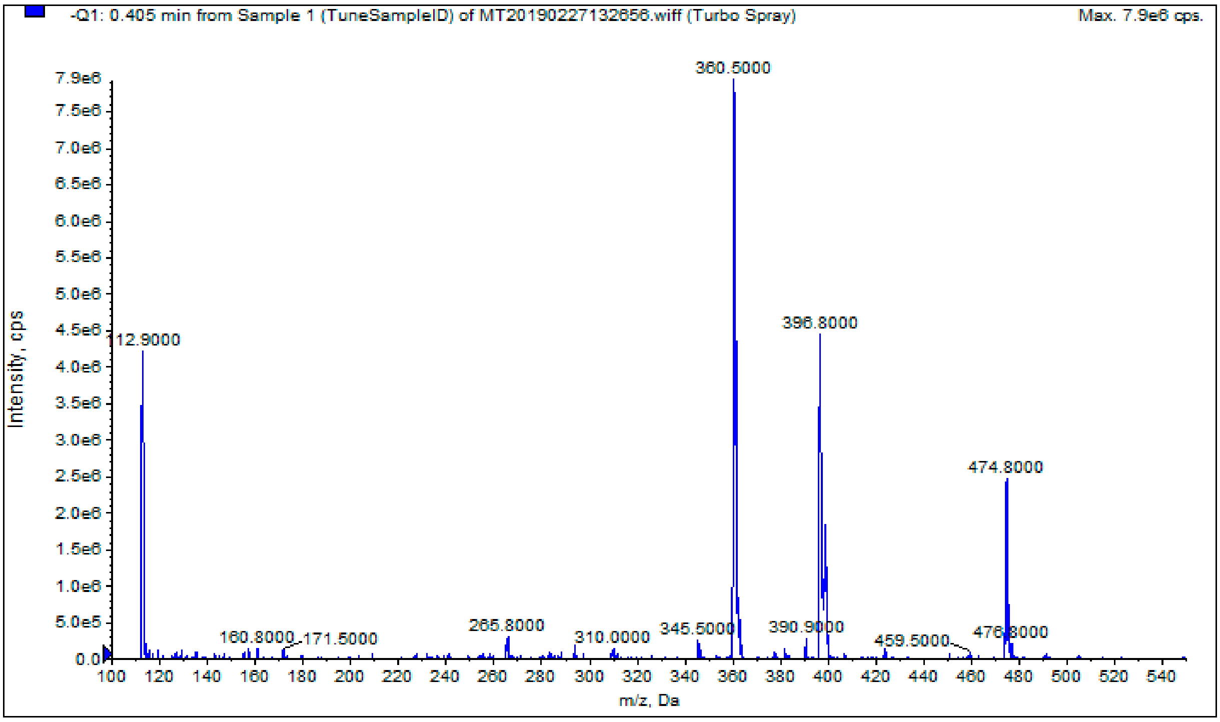

Figure S54. Mass spectrum of racemate **28** recorded in negative ionization mode. (solvent: CH_3CN)

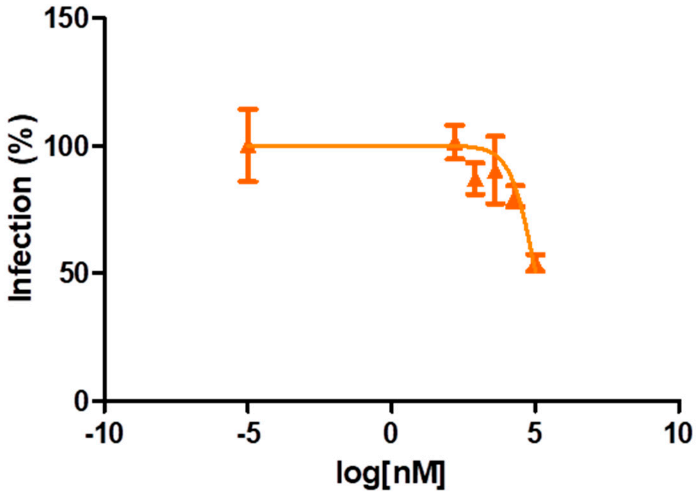

Figure S55. Compound **9** inhibits HIV-1 infection using the pseudotype virus assay at the non-cytotoxic concentration of 100 μM. Two independent experiments were performed in triplicates.

Table S1. Cytotoxicity of compounds **2-14** and **21-28** on U373-CD4-CCR5 cells, n=2. Compounds were tested up to 100 μM except for compound **9** whose cytotoxicity evaluation was repeated up to a maximum concentration of 500 μM. Both chemical strategies (B-ring saturation and 4′-oxime formation) allowed us to successfully decrease the cytotoxicity of protoflavones.

Compound	Compound type	Cytotoxic IC_{50} ± SEM [μM]
**2**	protoflavone	0.22 ± 0.003
**6**	protoflavone	2.09 ± 0.13
**7**	protoflavone	0.25 ± 0.002
**8**	protoflavone	0.67 ± 0.03
**9**	tetrahydroprotoflavone	> 500
**10**	tetrahydroprotoflavone	> 100
**11**	tetrahydroprotoflavone	> 100
**12**	tetrahydroprotoflavone	> 100
**13**	tetrahydroprotoflavone	> 100
**14**	tetrahydroprotoflavone	~ 6.37
**21**	dihydroprotoflavone 4′-oxime	> 100
**22**	dihydroprotoflavone 4′-oxime	> 100
**23**	dihydroprotoflavone 4′-oxime	~ 89.45
**24**	dihydroprotoflavone 4′-oxime	> 100
**25**	dihydroprotoflavone 4′-oxime	53.88 ± 0.35
**26**	dihydroprotoflavone 4′-oxime	> 100
**27**	tetrahydroprotoflavone 4′-oxime	> 100
**28**	tetrahydroprotoflavone 4′-oxime	27.02 ± 8.4
